# Two *Arabidopsis* Homologs of Human Lysine-Specific Demethylase Function in Epigenetic Regulation of Plant Defense Responses

**DOI:** 10.3389/fpls.2021.688003

**Published:** 2021-06-14

**Authors:** Seong Woo Noh, Ri-Ra Seo, Hee Jin Park, Ho Won Jung

**Affiliations:** ^1^Department of Applied Bioscience, Dong-A University, Busan, South Korea; ^2^Institute of Agricultural Life Science, Dong-A University, Busan, South Korea; ^3^Department of Molecular Genetics, Dong-A University, Busan, South Korea

**Keywords:** *Arabidopsis*, defense priming, epigenetic regulation, histone methylation, immunity, WRKY transcription factors

## Abstract

Epigenetic marks such as covalent histone modification and DNA methylation are crucial for mitotically and meiotically inherited cellular memory-based plant immunity. However, the roles of individual players in the epigenetic regulation of plant immunity are not fully understood. Here we reveal the functions of two *Arabidopsis thaliana* homologs of human lysine-specific demethylase1-like1, LDL1 and LDL2, in the maintenance of methyl groups at lysine 4 of histone H3 and in plant immunity to *Pseudomonas syringae* infection. The growth of virulent *P. syringae* strains was reduced in *ldl1* and *ldl2* single mutants compared to wild-type plants. Local and systemic disease resistance responses, which coincided with the rapid, robust transcription of defense-related genes, were more stably expressed in *ldl1 ldl2* double mutants than in the single mutants. At the nucleosome level, mono-methylated histone H3K4 accumulated in *ldl1 ldl2* plants genome-wide and in the mainly promoter regions of the defense-related genes examined in this study. Furthermore, *in silico* comparative analysis of RNA-sequencing and chromatin immunoprecipitation data suggested that several WRKY transcription factors, *e.g*., WRKY22/40/70, might be partly responsible for the enhanced immunity of *ldl1 ldl2*. These findings suggest that LDL1 and LDL2 control the transcriptional sensitivity of a group of defense-related genes to establish a primed defense response in *Arabidopsis*.

## Introduction

Plants have evolved various disease resistance responses and the plasticity of these responses helps ensure plant survival in the face of biotic stress. Plant immunity is divided into two categories based on the types of immune receptors employed (Jones and Dangl, 2006; Han and Jung, 2013). Microbe/pathogen-associated molecular pattern (MAMP/PAMP, hereafter MAMP)-triggered immunity (PTI) is initiated by pattern recognition receptors (PRRs) located on the plasma membrane that recognize MAMPs. Effector-triggered immunity (ETI), which is controlled by intracellular Nucleotide-binding Oligomerization Domain (NOD)-like receptors (NLRs), also known as plant disease resistance (R) proteins, is accompanied by a hypersensitive response (HR) (Chisholm et al., 2006; Jones and Dangl, 2006). Additionally, since PTI and ETI are associated with each other, the former can intensify the latter and *vice versa* (Qi et al., 2011; Jung et al., 2020; Ngou et al., 2021; Yuan et al., 2021). Local disease resistance governed by PRRs and NLRs also induces systemic acquired resistance (SAR), which effectively restrains the colonization of pathogens throughout most of the plant in the face of subsequent pathogen infection (Durrant and Dong, 2004; Fu and Dong, 2013). These different immune responses require specialized signaling networks and share conserved defense-related signaling and responses, including rapid, robust transcriptional changes in local and/or systemic tissues during infection (Tsuda et al., 2009; Tsuda and Katagiri, 2010; Gruner et al., 2013; Tsuda and Somssich, 2015).

Immune signaling can also induce cellular memory, which helps prime the plant to respond to future infections. Cellular memory is inherited both mitotically and meiotically from parental cells to daughter cells and leads to phenotypic variation in plants by regulating gene expression (Saze, 2008; Holeski et al., 2012). Differential gene expression, which leads to epigenetic variation in eukaryotes, is controlled by DNA methylation and several covalent modifications on the N-terminal tails of histone proteins such as acetylation, methylation, ubiquitination, phosphorylation, ADP-ribosylation, and sumoylation (Pikaard and Mittelsten Scheid, 2014; Ramirez-Prado et al., 2018). These chromatin modifications affect the transcriptional state of genes associated with specific changes.

‘Defense priming’ (such as SAR) is an adaptive strategy that fosters a faster and stronger defense response against subsequent challenge (Conrath et al., 2006, 2015; Beckers et al., 2009; Jung et al., 2009; Conrath, 2011). The immunization of the local leaves of plants with SAR-inducing stimuli encourages chromatin remodeling in distal systemic leaves (Jaskiewicz et al., 2011; Luna et al., 2012; Ramirez-Prado et al., 2018). For example, histone H3 and H4 lysine methylation (especially di- and tri-methylation) and acetylation on the promoter regions of *WRKY6*/*29*/*53* were induced in distal leaves after local infection with *Pseudomonas syringae* pv. *maculicola*. These modifications act as histone memory to help plants adapt to subsequent stresses. Hence, these genes are rapidly transcribed in the distal leaves of plants exposed to a second challenge (Jaskiewicz et al., 2011).

Induced resistance is epigenetically heritable in plants: disease-exposed plants can produce progeny that are primed for pathogen infection. In fact, the increased expression of salicylic acid (SA)-induced defense genes, such as *PATHOGENESIS-RELATED PROTEIN1* (*PR1*) and *WRKY6/53/70*, is correlated with specific histone modifications (such as H3K6ac and H3K27me) at the promoter regions of these genes in progeny from pathogen-infected plants, suggesting that plants memorize changes in histone marks in a transgenerational manner (Luna et al., 2012). Furthermore, open chromatin regions were identified in systemic leaves of *Arabidopsis* following challenge infection and were used to isolate SAR regulators (Baum et al., 2019). Repetitive exposure to environmental stresses also induces changes to histone modifications, which confer resistance/tolerance to biotic and abiotic stress in plants (Singh P. et al., 2014; Brzezinka et al., 2016; Raxwal et al., 2020). Moreover, the failure to add specific histone modifications perturbs resistance to (hemi)biotrophs and necrotrophs (reviewed in Ding and Wang, 2015), indicating that the covalent modification of histone proteins is crucial for plant immunity.

Histone methylation and demethylation primarily occur at specific lysine (Lys, K) residues on histone H3 and H4 proteins (H3K4, H3K9, H3K27, H3K36, H3K79, and H4K20) and affect the transcription of target genes (Hyun et al., 2017). In animal cells, human lysine-specific histone demethylase1 (LSD1), also known as lysine-specific demethylase 1A (KDM1A), removes mono- and di-methyl groups from the lysine residues of histone H3, specifically H3K4 and H3K9, and participates in various biological processes, *e.g.*, cell proliferation and tumor development (Shi et al., 2004; Chen et al., 2012). Therefore, human LSD1 is a proposed target for therapeutic purposes (Ellis and Loda, 2018; Tu et al., 2020). In plants, methyl groups of H3K4 are erased by Jumonji C domain-containing demethylases and plant LSD1-like (LDL) proteins (Spedaletti et al., 2008; Martignago et al., 2019).

The *Arabidopsis thaliana* genome contains four *LDL* genes [*At1g62830* (*LDL1*), *At3g13682* (*LDL2*), *At4g16310* (LDL3), and *At3g10390* (*LDL4*)], which are engaged in flowering time control, circadian clock regulation, homologous recombination repair, hormone responses, and systemic resistance (Jiang et al., 2007; Krichevsky et al., 2009; Singh et al., 2012, 2013; Shafiq et al., 2014; Zhao et al., 2015; Hung et al., 2018, 2019; Hirakawa et al., 2019; Martignago et al., 2019). For example, *Arabidopsis* LDL1/SWIRM domain PAO PROTEIN1 (LDL1/SWP1) represses the expression of *FLOWERING LOCUS C* (*FLC*), encoding a floral repressor, and *LATERAL ROOT PRIMODIUM1*, encoding a transcriptional activator, to promote auxin homeostasis-regulated gene expression in root primordia during early root development. Accordingly, the recessive *ldl1* mutants exhibit late flowering and enhanced root elongation and lateral root formation (Jiang et al., 2007; Singh et al., 2012, 2020; Shafiq et al., 2014). LDL1 also regulates seed dormancy by controlling the expression of *DELAY OF GERMINATION1*, encoding a regulator of primary dormancy and the abscisic acid (ABA) signaling pathway (Zhao et al., 2015). LDL1 also controls the dissociation of RAD54 from damaged DNA sites by recognizing H3K4me2 during homologous recombination repair and maintains gene stability and integrity (Hirakawa et al., 2019). LDL2 regulates primary seed dormancy and the circadian clock in cooperation with LDL1 (Zhao et al., 2015; Hung et al., 2018, 2019). Interestingly, LDL4/FLOWERING LOCUS D/REDUCED SYSTEMIC IMMUNITY1 (LDL4/FLD/RSI1) positively regulates systemic resistance against *Pseudomonas* infection, and *ldl4*/*fld*/*rsi1* mutants show hyper-susceptibility to necrotrophic fungal infection in local tissues (Singh et al., 2013, 2019; Singh P. et al., 2014). These findings suggest that other *Arabidopsis* LDL proteins also participate in plant immunity.

Here we uncovered the roles of *Arabidopsis* LDL1 and LDL2 in plant immune responses against *Pseudomonas* infection. Loss-of-function mutations in *LDL1* and *LDL2*, which act in a partially redundant manner, led to local and systemic disease resistance to phytopathogenic bacterial infection, along with increased expression of defense-related genes involved in SA- and MAMP-dependent signaling following *Pseudomonas* infection. *In silico* comparative analysis of RNA-sequencing (RNA-seq) and previously published chromatin immunoprecipitation (ChIP)-seq data identified 39 differentially expressed genes (DEGs) whose nucleosome modifications might be controlled by LDL1 and LDL2. Monomethylated H3K4 (H3K4me1) strongly accumulated in several defense-related genes in *ldl1 ldl2* double mutants compared with wild-type (WT) plants. Our findings suggest that LDL1 and LDL2 are internal targets that establish primed defense responses in *Arabidopsis*.

## Materials and Methods

### Plant Materials and Growth

The *Arabidopsis thaliana* mutants used in this study are as follows: *ldl1-1* (*ldl1*, Salk_048276), *ldl1-2* (Salk_034869), *ldl1-3* (Salk_108984), *ldl2-1* (*ldl2*, Salk_135831), *ldl2-2* (Salk_138820), *ldl3* (SALK_146733), and *ldl4*/*fld* (Salk_015053). All mutants are in the Columbia-0 (Col-0) background. The *ldl1 ldl2* double mutant was generated by crossing *ldl1-1* to *ldl2-1*. *Arabidopsis* plants were grown in soil (Nongwoo Bio) under neutral day conditions (12 h light/12 h dark cycles, relative humidity 60–70%, 120 μmol m^–2^s^–1^, 22 ± 1°C) in a walk-in growth chamber (Jung et al., 2020).

For the root growth assay, 5-day-old *Arabidopsis* seedlings grown on half-strength Murashige and Skoog (MS) medium were transferred to MS medium supplemented with 25 and 50 μM salicylic acid (SA, Sigma-Aldrich). The seedlings were grown in medium under neutral day conditions for 2 weeks, and root lengths were measured using the Fiji program (Schneider et al., 2012).

### Bacterial Strains and Inoculation

*Pseudomonas syringae* pv. *maculicola* ES4326 (*Psm*ES4326) [newly classified as *P. cannabina* pv. *alisalensis* (Bull et al., 2010)] and *P. syringae* pv. *tomato* DC3000 (*Pst*DC3000) were used as virulent *Pseudomonas* strains. The avirulent derivatives of *P. syringae* were *Psm*ES4326 carrying *AvrRpt2* (*Psm*ES4326/*AvrRpt2*, DG6) and *Psm*ES4326 carrying *AvrRpm1* (*Psm*Es4326/*AvrRpm1*, DG34) (Guttman and Greenberg, 2001). As virulence-deficient and attenuated strains of *P. syringae*, we employed *Pst*DC3000 *hrcC*^–^ and *Pst*DC3000△*AvrPto*△*AvrPtoB*, respectively (Roine et al., 1997; Guttman et al., 2002; Lin and Martin, 2005) (Supplementary Table 1).

Bacterial strains were freshly prepared in King’s B medium supplemented with the appropriate antibiotics (Supplementary Table 1) and diluted to various concentrations in 10 mM MgSO_4_ as follows: OD_600_ = 0.01 for immunization with an avirulent pathogen (*Psm*ES4326/*AvrRpt2*, DG6) and pathophysiological studies (*Psm*ES4326 and *Pst*DC3000); OD_600_ = 0.0001 to test disease responses (all strains). The strains were inoculated on three fully expanded leaves of 4-week-old *Arabidopsis* plants to evaluate pathophysiological responses and to count bacterial growth in leaf discs. The number of bacteria in infected leaves was determined 3 d after inoculation using a typical serial dilution method, and the infected leaves were photographed on the same day.

### RNA Extraction and Quantitative RT-PCR (qRT-PCR) Analysis

Leaves of 4-week-old *Arabidopsis* (∼0.1 g) plants were harvested at the indicated time points after *Pseudomonas* inoculation. Total RNA was extracted from the samples using TRIzol reagent (Thermo Fisher Scientific), and contaminating genomic DNA was removed using TURBO DNase (Ambion). First-strand cDNA was synthesized from 5 μg of total RNA using SuperScript-II Reverse Transcriptase (Thermo Fisher Scientific) according to the manufacturer’s instructions. Quantitative PCR (qPCR) was performed using SYBR Premix Ex Taq (TaKaRa Bio) and a CFX384 Real-time PCR Detection System (Bio-Rad). The cycling conditions were 95°C for 10 min, and 50 cycles of 95°C for 5 s, 60°C for 10 s, and 72°C for 35 s. *ACTIN2* (At3g18780) was used as the reference gene to normalize transcript levels. Relative expression levels were analyzed using the comparative cycle threshold (ΔΔCt) method (Livak and Schmittgen, 2001) and are shown as mean ± SD (standard deviation). All experiments were performed with at least three biological replicates with two or three technical repeats unless otherwise noted. Asterisks indicate statistically significant differences from WT plants (^∗^*p* < 0.05, ^∗∗^*p* < 0.01, two-tailed Student’s *t*-test). The oligonucleotide sequences of the primers are shown in Supplementary Table 2.

### Transcriptome Analysis

Total RNA was isolated from the leaves of 3-week-old WT (Col-0) and *ldl1 ldl2* plants grown under neutral day conditions, using TRIzol reagent. Precipitated and dissolved total RNA was cleaned using an RNeasy spin column (Qiagen). After confirming the purity of the RNA (Bioanalyzer, Agilent), the total RNA was used to construct an mRNA sequencing library using a TruSeq Stranded mRNA Sample Preparation Kit according to the manufacturer’s instructions (Illumina). Each library (from two biological replicates per genotype) was subjected to 100 bp paired-end sequencing on the HiSeq 2000 platform (Illumina), as described previously (Jung et al., 2020). The RNA-seq data were processed with TopHat2 and Bowtie2 (Trapnell et al., 2009; Langmead and Salzberg, 2012). To quantify the total transcript mass in fragments per kilobase of transcript per million mapped reads (FPKM), data from biological replicates of WT and *ldl1 ldl2* plants were separately aligned with the *A. thaliana* TAIR10 gene model using the Cufflinks package (Trapnell et al., 2010). RNA-seq data have been deposited in the National Agricultural Biotechnology Information Center (NABIC)^1^ under accession numbers NN-1560, NN-1561, NN-1578, and NN-1580 and at the GEO datasets in National Center for Biotechnology Information (NCBI) (GSE171433).

### Protein Extraction and Immunoblot Analysis

Total proteins were extracted from 4-week-old *Arabidopsis* leaves using protein extraction buffer (20 mM Tris-HCl [pH 7.5], 150 mM NaCl, 1 mM EDTA, 0.1% [v/v] Triton X-100, 0.1% [w/v] SDS, 5 mM DTT and proteinase inhibitors [Pierce Protease inhibitor, Thermo Fisher Scientific]) (Jung et al., 2020). SDS-polyacrylamide gel electrophoresis using Tris-glycine electrophoresis buffer and immunoblot analysis were carried out as described (Green and Sambrook, 2012). The antibodies used in this study are as follows: α-BAK1 (AS12 1858, Agrisera), α-H3K4me1 (PA5-17418, Invitrogen), α-H3K4me2 (701764, Invitrogen), α-H3K4me3 (PA5-17420, Invitrogen), and α-H3 (AS10 710, Agrisera) antibodies. The signal was visualized with SuperSignal Chemiluminescent Substrate (Thermo Fisher Scientific).

### SA Measurement and Staining of Deposited Callose

Free SA levels in infected leaves were measured using a high-performance liquid chromatograph coupled with a fluorescence detector (Agilent 1100) as described previously (Seskar et al., 1998; Jung et al., 2009).

Four-week-old *Arabidopsis* plants grown under neutral day conditions were inoculated with 10 mM MgSO_4_ and *Pst*DC3000 (OD_600_ = 0.01), and 10 infected leaves were detached at 24 h post-inoculation (hpi). The leaf tissues were submerged in destaining solution (acetic acid:ethanol = 1:3) overnight. After rinsing the cleared leaves with 150 mM K_2_HPO_4_ for 30 min, the leaves were incubated in 0.01% aniline blue solution in 150 mM K_2_HPO_4_ for 2 h (Schenk et al., 2014). Deposited callose in infected leaves was detected by confocal microscopy (LSM 700 laser scanning confocal microscope, Carl-Zeiss), and the callose deposits were quantified using the Fiji program (Schneider et al., 2012).

### Chromatin Immunoprecipitation (ChIP) and qPCR

Leaf tissues were collected from 3-week-old WT and *ldl1 ldl2* plants grown in a walk-in growth chamber and used to extract nuclei, as previously described (Jaskiewicz et al., 2011; Jung et al., 2020). Extracted nuclei were homogenized in nuclei lysis buffer [50 mM Tris-HCl (pH 8.0), 10 mM EDTA, 1% (w/v) SDS, and proteinase inhibitors (Roche)], and the resulting chromatin was sheared by sonication to obtain fragment sizes of 200–800 bp (Bioruptor, Diagenode). ChIP was performed using α-H3K4me1, α-H3K4me2, and α-H3K4me3 antibodies (Invitrogen) according to the manufacturer’s instructions (Pierce Agarose ChIP Kit, Thermo Fisher Scientific). The enrichment of modified histone proteins in the genes of interest was determined by qPCR (Jung et al., 2020) and calculated by the percent input method (Lin et al., 2012). The primers used in this study are listed in Supplementary Table 2.

## Results

### The *ldl1 ldl2* Double Mutant Shows Restricted Multiplication of Virulent *Pseudomonas* Strains, but Not Avirulent or Attenuated Derivatives of *Pseudomonas*

To explore whether *Arabidopsis LDL* genes are involved in plant immune responses, we examined the mRNA expression pattern of *LDL*s upon bacterial pathogen infection using the visualized meta-analysis database eFP^2^ (Winter et al., 2007). Leaves of 4-week-old *Arabidopsis* WT plants were inoculated with 10 mM MgSO_4_ (mock, M), an avirulent pathogen (*Psm*ES4326/*AvrRpt2*, A), or a virulent pathogen (*Psm*ES4326, V). The transcript levels of the *LDL*s were examined using ATH1 GeneChip at the indicated hours after inoculation and normalized by *ACTIN2* (Supplementary Figure 1A). Mock (10 mM MgSO_4_) treatment did not affect the expression of any of the four *LDL* homologs: *LDL1*, *LDL2*, *LDL3*, and *LDL4*/*FLD*. Both avirulent and virulent bacterial infection had little effect on the expression of *LDL1* and *LDL4*/*FLD*. Infection with the avirulent strain *Psm*ES4326/*AvrRpt2* induced *LDL2* expression and reduced *LDL3* expression. However, since these differences in expression were within 1.5-fold and 0.5-fold, these findings suggest that the expression of *LDL*s in local leaves is not altered by bacterial infection.

We also examined the expression of the *LDL*s in the distal systemic leaves of plants immunized with the SAR-inducing *Pseudomonas* strain *Psm*ES4326/*AvrRpt2* (DG6) (Supplementary Figure 1B). At 2 days after local infection (F) with the avirulent strain *Psm*ES4326/*AvrRpt2*, we inoculated distal leaves (S) with the virulent *Psm*ES4326 strain and examined the expression of the *LDL*s by qRT-PCR (Supplementary Figure 1C). The *LDL* genes in systemic leaves were not significantly up- or down-regulated compared to non-infected leaves, as their changes in expression were within 0.5-fold. However, at 10 h after challenge-inoculation, *LDL1* and *LDL3* expression significantly decreased in systemic leaves compared to the control.

Next, we tested the disease resistance responses of *ldl* mutants against *Psm*ES4326 infection. The *ldl4*/*fld*/*rsi1* mutants are defective in systemic resistance, but they still show WT-like local susceptibility to *Pseudomonas* infection (Singh et al., 2013). Under the experimental conditions we used to identify mutants exhibiting enhanced disease resistance or susceptibility against *Pseudomonas* infection, the titers of the virulent *Psm*ES4326 strain decreased in *ldl1* and *ldl2*, but not in *ldl3* or *ldl4*/*fld*, compared to WT plants (Supplementary Figure 1D). To confirm the enhanced disease resistance of the *ldl1* and *ldl2* mutants, we inoculated different mutant alleles with *Psm*ES4326 and *Pst*DC3000. The mutants were resistant to both of these virulent *Pseudomonas* strains (Supplementary Figure 1E). These results indicate that both LDL1 and LDL2 suppress the immune response against *Pseudomonas* infection.

**FIGURE 1:**
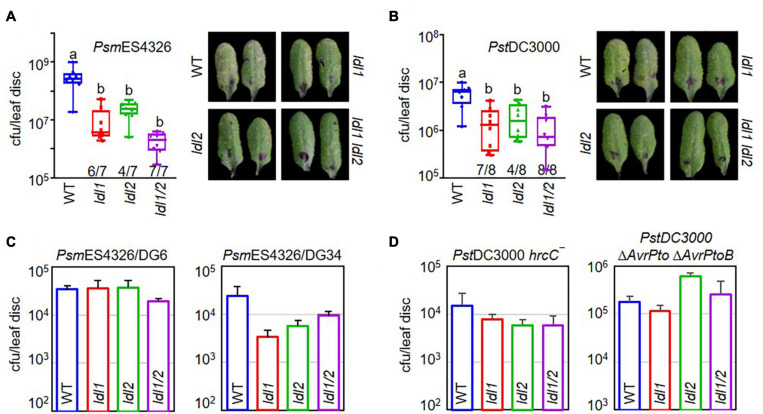
The hyper-resistance response of *ldl1 ldl2* double mutants to virulent *Pseudomonas* infection is more stable than that of the single mutants. **(A**,**B)** The growth of *P. syringae* pv. *maculicola* ES4326 (*Psm*ES4326, OD_600_ = 0.0001) **(A)** and *P. syringae* pv. *tomato* DC3000 (*Pst*DC3000, OD_600_ = 0.0001) **(B)** in WT, *ldl1*, *ldl2*, and *ldl1 ldl2* plants at 3 days post inoculation (dpi). The denominators and numerators under the box plots indicate the number of total repeats and the number of trials showing differences from WT, respectively. Box plots show the minimum, first quartile, median, third quartile, and maximum values. The right panels show disease symptoms of each genotype caused by *P*. *syringae* infection. **(C**,**D)** WT-like disease responses of single and double mutants against infection with different avirulent derivatives of *Psm*ES4326 carrying *AvrRpt2* (*Psm*ES4326/DG6, OD_600_ = 0.0001) and *AvrRpm1* (*Psm*ES4326/DG34, OD_600_ = 0.0001) **(C)** and attenuated mutants of *Pst*DC3000 *hrcC*^–^ (OD_600_ = 0.001) and *Pst*DC3000 Δ*AvrPto* Δ*AvrPtoB* (OD_600_ = 0.001) **(D)**. The area of a leaf disc is 0.78 cm^2^. Bar indicates the average ± standard error (SEM) (*p* < 0.05, ANOVA-Tukey, *n* = 8). The experiments were repeated 3, 6, and 3 times for the avirulent derivatives (*Psm*ES4326/DG6 and *Psm*ES4326/DG34), *Pst*DC3000 *hrcC*^–^, and *Pst*DC3000 Δ*AvrPto* Δ*AvrPtoB*, respectively, with similar results.

To exclude the possibility of functional redundancy between LDL1 and LDL2, as these proteins share 53% identity and 68% similarity, we crossed *ldl1-1* with *ldl2-1* and tested the disease resistance of the *ldl1 ldl2* double mutants against infection with *Psm*ES4326 and *Pst*DC3000 (Figures 1A,B). The *ldl1* and *ldl2* single mutants exhibited disease resistance 6 and 4 times out of 7 individual infections with *Psm*ES4326, respectively. The disease resistance response was more stable and firmly established in the *ldl1 ldl2* mutants (7 out of 7 replicates) than the single mutants, even though the extent of resistance observed in *ldl1 ldl2* was not significantly different from that of the single mutants (Figure 1A). We repeated the bacterial growth test in the *ldl1 ldl2* mutants after *Pst*DC3000 infection eight times. Like the stable resistance response against *Psm*ES4326, the double mutants were resistant to *Pst*DC3000 infection in all eight independent experiments compared to the WT (Figure 1B).

The multiplication of avirulent pathogens can be restrained via an ETI-mediated pathway involving R proteins and various essential components, such as NDR1 (NON-RACE SPECIFIC DISEASE RESISTANCE1) and EDS1 (ENHANCED DISEASE SUSCEPTIBILITY1) (Parker et al., 1996; Century et al., 1997; Aarts et al., 1998; Falk et al., 1999). Therefore, we evaluated the resistance response of the *ldl1 ldl2* mutants to avirulent pathogen infection to examine whether LDL1 and LDL2 are engaged in ETI (Figures 1C,D). *Psm*ES4326/*AvrRpt2* (DG6) and *Psm*ES4326/*AvrRpm1* (DG34) were inoculated into the leaves of WT, *ldl1*, *ldl2*, and *ldl1 ldl2* plants. All plants with mutations in *LDL1* or *LDL2* showed comparable (similar) disease susceptibility to WT plants, suggesting that LDL1 and LDL2 are not involved in *RPS2-* and *RPM1*-mediated ETI in *Arabidopsis* (Figure 1C).

Phytopathogenic *P. syringae* uses the Type III secretion system (T3SS) to deliver effector proteins into plant cells to manipulate and/or inhibit host proteins (Collmer et al., 2002; Lindeberg et al., 2009, 2012). Thus, attenuated derivatives of *P. syringae* with defects in the T3SS machinery or effectors fail to colonize plants, since these strains cannot overcome the basal immune response initiated by the PRR extracellular immune receptors (Deng et al., 2017). Both the *ldl* single and double mutants showed WT-like resistance against infection with two attenuated *P. syringae* strains, *Pst*DC3000 *hrcC*^–^ and *PstDC3000 ΔAvrPto ΔAvrPtoB* (Figure 1D). These results demonstrate that individual mutations of *LDL1* and *LDL2* confer resistance to infection with virulent *Pseudomonas* and that each gene can compensate for the other.

### SA- and MAMP-Responsive Genes Are Rapidly Transcribed in *ldl1*, *ldl2*, and *ldl1 ldl2* Mutants After *Pseudomonas* Infection

**FIGURE 2:**
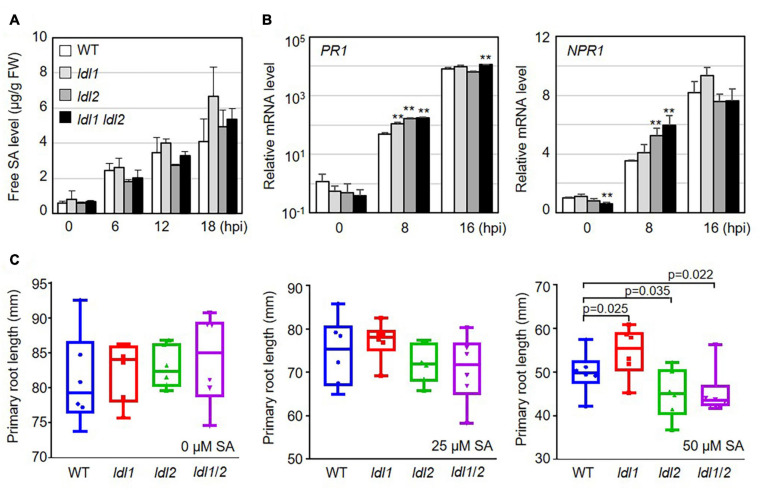
Transcription of salicylic acid (SA)-dependent signaling genes is more rapidly induced in *ldl* mutants than in WT plants after *Pseudomonas* infection. **(A)** Free SA levels in the leaves of *Arabidopsis* plants infected with the virulent *Psm*ES4326 strain (OD_600_ = 0.01). The experiments were repeated three times with similar results. **(B)** Rapid expression of SA-responsive genes in *Psm*ES4326 (OD_600_ = 0.01)-infected leaves of *ldl1*, *ldl2*, and *ldl1 ldl2* plants. The averages ± SD (standard deviation) were plotted (**p < 0.01, two-tailed Student’s *t*-test, *n* = 3). Five biologically independent experiments with 3 technical repeats were performed, with similar trends, as shown in Supplementary Figure 2. **(C)** Primary root growth of WT, *ldl1*, *ldl2*, and *ldl1 ldl2* plants grown in half-strength MS medium supplemented with 25 and 50 μM SA for 14 days. Box plots show the minimum, first quartile, median, third quartile, and maximum values. Each dot represents the average of independent biological replicates (*n* = 6), with fives samples per individual replicate (*p*-Values from two-tailed Student’s *t*-test, *n* = 6).

SA accumulation upon bacterial infection occurs as part of the plant immune response to (hemi)biotrophic microbes. To investigate whether the resistance responses of the *ldl* mutants function via SA-dependent immunity, we measured free SA levels in the leaves of WT and mutant plants infected with *Psm*ES4326. Following pathogen infection, free SA accumulated in infected leaves regardless of plant genotype, and these levels did not significantly differ in the different genotypes (Figure 2A).

We then measured the transcript levels of genes essential for SA-dependent immunity, including the following: *PR1* (SA responsiveness), *NON-EXPRESSOR OF PR1* (*NPR1*, SA perception/responsiveness), *AGD2-LIKE DEFENSE1* (*ALD1*, SA regulation), and *WRKY70* (encoding an activator of SA-dependent defense genes and a modulator of antagonistic interactions between SA and JA signaling). All genes tested in this study were actively transcribed in the *ldl* single and double mutants, compared with WT plants, at the early phase (8 hpi) after local infection of *Psm*ES4326 (Figure 2B and Supplementary Figure 2). To avoid overestimating the expression levels, we independently repeated the experiments five times, as summarized in Supplementary Figure 2. The transcript levels of the genes before infection (0 hpi) and at 16 hpi were not consistent among the experiments. However, despite these experimental variables, mRNA levels were higher in the *ldl* mutants, especially the *ldl1 ldl2* double mutants, than in WT plants at 8 hpi (Figure 2B and Supplementary Figure 2).

The application of high concentrations of exogenous SA to *Arabidopsis* seedlings retards primary root elongation (Pasternak et al., 2019). Thus, we expected that the primary roots of the *ldl* mutants would be shorter than those of WT plants. To exclude a false positive effect due to the increased seed dormancy of *ldl1 ldl2* mutants (Zhao et al., 2015), we transferred 5-day-old seedlings grown on 1/2 MS medium to 1/2 MS medium containing different concentrations of SA (0, 25, and 50 μM) and grew the seedlings under neutral day conditions for 14 days (12-h light/12 h dark). Primary root growth on 0 and 25 μM SA was not altered by the mutation of *LDL1* and/or *LDL2* (left and middle panels in Figure 2C). However, at 50 μM SA, the primary roots were significantly longer in *ldl1* seedlings but shorter in *ldl2* and *ldl1 ldl2* seedlings compared to the WT (right panel in Figure 2C). These findings demonstrate that the *ldl1 ldl2* mutants are hypersensitive to SA-dependent signaling, as confirmed by the finding that the expression of SA-responsive genes occurred rapidly in these plants during the early infection phase (Figure 2B and Supplementary Figure 2).

**FIGURE 3:**
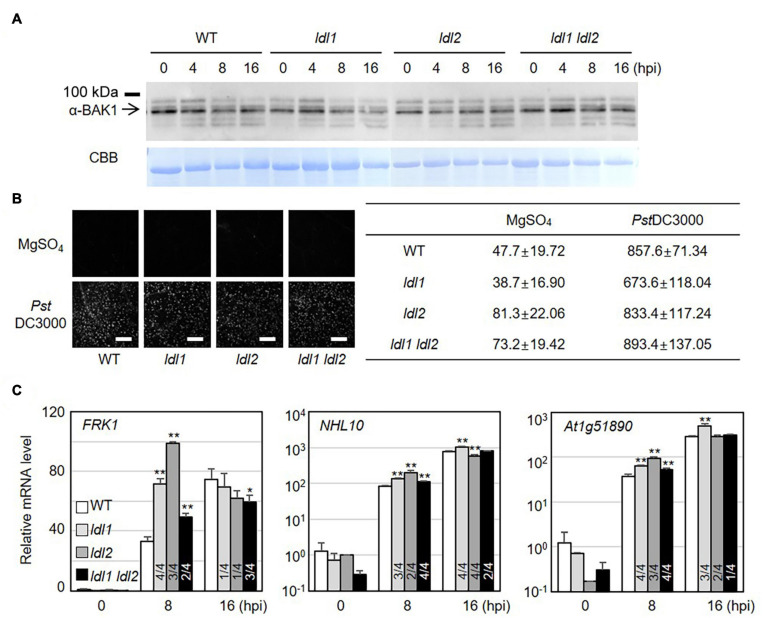
Transcript levels of MAMP-responsive genes increase in *ldl* mutants after *Pseudomonas* infection. **(A)** BAK1 protein abundance in WT, *ldl1*, *ldl2*, and *ldl1 ldl2* during *Pst*DC3000 infection (OD_600_ = 0.01). **(B)** Callose deposition in *Arabidopsis* leaves infiltrated with *Pst*DC3000 at 24 h post-inoculation (hpi). Equivalent volumes of 10 m MgSO_4_ were used for mock conditions. Left panel: representative portions of leaves from WT, *ldl1*, *ldl2*, and *ldl1 ldl2* plants stained with aniline blue to visualize deposited callose. Right panel: average ± SD of the number of callose deposits per field (*n* = 10) (Scale bars are 300 μm). No significant differences from WT plants were observed when analyzed with a one-way ANOVA-Tukey test (*p* < 0.05). **(C)** Transcript levels of *FRK1*, *NHL10*, and *At1g51890* in local leaves of WT, *ldl1*, *ldl2*, and *ldl1 ldl2* plants at 0, 8, and 16 hpi with *Pst*DC3000 (OD_600_ = 0.01). Bar indicates the SD (*p < 0.05, **p < 0.01, two-tailed Student’s *t*-test, *n* = 3). The experiments were repeated 4 times; the numerators of the fractions below each plot indicate the number of trials showing differences from WT plants.

PTI represents a frontline defense barrier that protects plants from pathogen infection along with SA-related events. Since the growth rates of two different attenuated *P. syringae* strains in *ldl* mutants were similar to those in WT plants, we reasoned that the PTI response might not be dramatically altered in the *ldl* mutants. As expected, the protein abundance of BRASSINOSTEROID INSENSITIVE1-ASSOCIATED RECEPTOR KINASE1 (BAK1) and the amount of deposited callose in infected leaves of the mutants were comparable to those in WT plants (Figures 3A,B). Next, we examined the transcriptional regulation of MAMP-responsive genes in WT and mutant plants after *Pst*DC3000 infection. The transcript levels of NDR/HIN1-LIKE 10 (*NHL10*) and *At1g51890* (encoding a leucine-rich repeat protein kinase) increased at 8 hpi in the *ldl1* and *ldl1 ldl2* mutants, with similar results in three out of four independent experiments. At 8 hpi, *FLG22-INDUCED RECEPTOR-LIKE KINASE1* (*FRK1*) transcript levels in the *ldl1 ldl2* mutants were similar to or higher than those of WT plants (Figure 3C and Supplementary Figure 3). By contrast, at 16 hpi, the expression patterns of *FRK1*, *NHL10*, and *At1g51890* varied depending on the gene, plant genotype, or biological repeat, and therefore, it appears that LDL1 and LDL2 have little or no effect on regulating the transcription of these genes (Figure 3C and Supplementary Figure 3). These results indicate that the transcription of some MAMP-responsive genes, as well as SA-responsive genes, is more sensitive in the *ldl1 ldl2* mutants than the WT during the early phase of infection.

### *ldl1 ldl2* Mutants Show Stable Systemic Resistance Against Secondary *Pseudomonas* Infection

**FIGURE 4:**
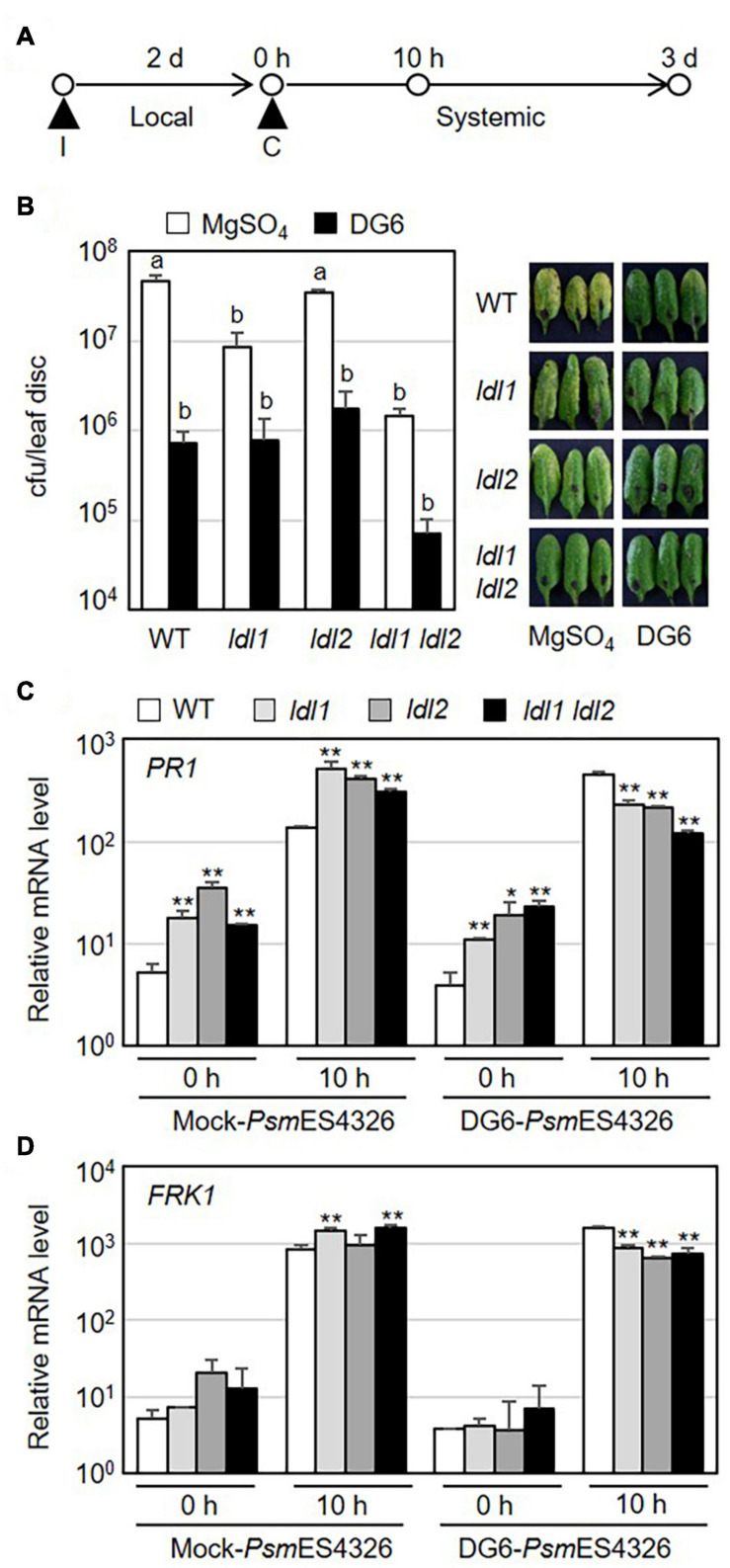
Systemic resistance is not further enhanced in *ldl1 ldl2* double mutants. **(A)** Schematic diagram of the timepoints for pre-immunization (I) with avirulent strain *Psm*ES4326/*AvrRpt2* (DG6) (OD_600_ = 0.01) or 10 mM MgSO_4_ (mock), the second challenge inoculation (C) with virulent *Psm*ES4326 (OD_600_ = 0.0001 or 0.01), and tissue samplings to evaluate the expression of defense-related genes and bacterial growth. **(B)** The growth of virulent *Psm*ES4326 in distal leaves of plants treated with 10 mM MgSO_4_ or avirulent *Psm*ES4326/*AvrRpt2* (DG6). Bars indicate SEM (*n* = 8). Different letters indicate significant differences (*p* < 0.05, one-way ANOVA-Tukey test). Photographs of infected leaves were taken at 3 days post-challenge inoculation. The experiments were repeated at least three times with similar results. **(C,D)** Transcript levels of *PR1* and *FRK1* in distal leaves of immunized plants before (0 h) and after challenge inoculation (10 h) with *Psm*ES4326 (OD_600_ = 0.01). Bar indicates the SD (*p < 0.05, **p < 0.01, two-way Student’s *t*-test, *n* = 3). The experiments were repeated twice with similar results (Supplementary Figure 4).

Specific covalent modifications of histone N-terminal tails, such as H3 and H4 acetylation and H3K4 methylation, reflect the establishment of systemic resistance (Jaskiewicz et al., 2011; Ding and Wang, 2015). As mentioned above, the *ldl1 ldl2* double mutants showed an enhanced disease resistance response against local infection by virulent *Pseudomonas* strains. To investigate whether the enhanced local resistance in the mutants leads to accelerated systemic resistance, we performed SAR assays in which plants (WT, *ldl1*, *ldl2*, and *ldl1 ldl2* plants) were exposed to a priming infection with avirulent strain *Psm*ES4326/DG6 in local leaves, followed 2 days later by challenge inoculation with *Psm*ES4326 in distal leaves (Figure 4A). The priming effect with successfully triggered systemic resistance was observed in WT and *ldl2* plants (Figure 4B). However, we noted that the local resistance of *ldl2* was not stable (Figures 1A,B). Bacterial growth and symptom development in distal leaves of *ldl1* and *ldl1 ldl2* mutants were also effectively restricted after challenge inoculation regardless of immunization (Figure 4B).

Systemic resistance usually correlates with stronger expression of defense-related genes in immunized plants than non-immunized plants (Conrath et al., 2015; Mauch-Mani et al., 2017; Jung et al., 2020). To test if the mutations of *LDL1* and *LDL2* lead to increased transcription of defense-related genes during SAR, we measured the mRNA levels of defense-related genes in distal leaves of immunized plants before and after challenge inoculation with *Psm*ES4326. The expression levels of *PR1*, *NPR1*, *WRKY70*, and *FRK1* did not consistently differ among WT and mutant plants before subsequent pathogen infection, although they were sometimes higher in mutant vs. WT plants (0 h in Figures 4C,D and Supplementary Figure 4). In the distal leaves of *Psm*ES4326/DG6-immunized plants, the expression patterns of these genes were also comparable (and sometimes even lower) in mutant vs. WT plants (10 h in Figures 4C,D and Supplementary Figure 4). However, mock-immunization in local leaves triggered strong transcription of these genes in mutant plants, especially *ldl1* and *ldl1 ldl2*, compared to WT plants (10 h in Figures 4C,D and Supplementary Figure 4). These results, together with the increased transcript levels in local infected leaves (Figure 2 and Supplementary Figure 2), indicate that the upregulation of defense-related genes reflects the enhanced disease resistance of the *ldl* mutants after pathogen infection.

### Several WRKYs Are Responsible for the Transcriptional Changes in *ldl1 ldl2*

To identify genes whose expression was affected by the simultaneous mutation of *LDL1* and *LDL2*, we compared the whole transcriptomes of *ldl1 ldl2* vs. WT plants. By analyzing mRNA-seq data from two biological replicates, we identified 273 DEGs in the *ldl1 ldl2* mutants (Supplementary Table 2). We performed Gene Ontology (GO) analysis of these DEGs to identify GO terms that are enriched among up- or downregulated DEGs in the *ldl1 ldl2* mutants (Mi et al., 2013). Of the 273 DEGs, 188 were successfully were mapped to GO IDs. A considerable portion of these DEGs (129 genes) appear to play roles in ‘response to stimulus’ (*p* < 1.08E-42, Fisher’s exact test). The top 10 biological processes included ‘cellular response to hypoxia,’ ‘responses to biotic and abiotic stress,’ ‘defense response,’ and ‘regulation of transcription’ (*p* < 1.E-04, Fisher’s exact test) (Supplementary Figure 5A). Two major functions of over-represented genes in *ldl1 ldl2* were ‘regulation of transcription’ and ‘protein binding’ (*p* < 1.E-04, Fisher’s exact test) (Supplementary Figure 5B). Similarly, 30 DEGs (*p* < 1.78E-07, Fisher’s exact test) and 6 DEGs (*p* < 2.51E-03, Fisher’s exact test) encode ‘transcription factors’ and ‘calmodulin-related calcium-binding proteins,’ respectively (Supplementary Figure 5C).

Previous microarray analysis identified 449 misregulated genes in the *ldl1 ldl2* double mutant (Berr et al., 2015). In addition, a more recent ChIP-seq analysis obtained from plants expressing *LDL1-GFP* (under its own promoter) revealed that LDL1 regulates the accumulation of methylated histone on the chromatin regions of 3,962 genes in the *Arabidopsis* genome (Hung et al., 2018). To narrow down LDL1 and LDL2 target genes in *Arabidopsis*, we compared these putative targets of LDL1 and LDL2 (Berr et al., 2015; Hung et al., 2018) with the 273 DEGs identified in the current study. Since the subset in common between the microarray data and RNA-seq data was too small to analyze, we focused on 39 DEGs that overlapped with LDL1 target genes identified by ChIP-seq (Table 1 and Supplementary Figure 5D). Since the well-known LDL1/LDL2-dependent genes *MADS AFFECTING FLOWERING 4* (*MAF4*) and *MAF5/AGAMOUS-LIKE68* (*AGL68*) (Berr et al., 2015; Hung et al., 2018) were identified in the *in silico* comparative analysis, we further analyzed these 39 DEGs to obtain clues to help explain the phenotypes of the *ldl1 ldl2* mutants.

**TABLE 1:** List of DEGs in *ldl1 ldl2* mutants overlapping with putative LDL1 target genes described by Hung et al. (2018).

Gene locus	Gene name/symbol	Gene description	Protein class	GO biological process	GO molecular process
At1g20510	*OPCL1*	OPC-8:0 CoA ligase1	Ligase	Response to bacterium, response to JA biosynthetic process	4-coumarate-CoA ligase activity
At1g33760	*ERF022*	DREB subfamily A-4 of ERF/AP2 transcription factor family	Transcription factor	Regulation of transcription, DNA-templated	DNA-binding transcription factor activity
At1g55450	At1g55450	S-adenosyl-L-methionine-dependent methyltransferases superfamily protein	-	-	-
At1g61340	*F-BOX STRESS INDUCED1*	F-box family protein	E3 ubiquitin ligase	Response to bacterium, response to SA, JA, ethylene, and ABA	-
At1g62480	At1g62480	Vacuolar calcium-binding protein-like protein	-	Response to cadmium ion	-
At1g72910	At1g72910	Toll-Interleukin-Resistance domain-containing protein	-	Response to bacterium	-
At1g74450	At1g74450	BPS1-like protein (DUF793)	-	Pollen development	-
At1g77960	*RESPONSE TO GLF1 OVEREXPRESSION*	Repressor ROX-1 like protein	-	-	-
At1g80840	*WRKY40*	WRKY DNA-binding protein 40	Transcription factor	Defense response to bacterium, regulation of defense response	DNA-binding transcription factor activity
At2g01180	*LPP1/PAP1*	Phosphatidic acid phosphatase 1	Phosphatase	Cellular response to hypoxia, response to UV-B	Phosphatidate phosphatase activity
At2g15390	*FUT4*	Fructosyltransferase 4	Transferase	Response to salt stress, protein glycosylation	Transferase activity, transferring glycosyl groups
At2g22500	*PUMP5/DIC1/UCP5*	Mitochondrial uncoupling protein 5	Secondary carrier transporter	Cellular response to hypoxia, oxaloacetate transport	Dicarboxylic acid transmembrane transporter activity
At2g30250	*WRKY25*	WRKY DNA-binding protein 25	Transcription factor	Response to osmotic stress, cellular response to heat	DNA-binding transcription factor activity
At2g46510	*AIB/BHLH17/JAM1*	ABA-inducible bHLH-type transcription factor	Transcription factor	Response to wounding, response to abscisic acid	DNA-binding transcription factor activity
At3g01830	*CML40*	Calcium-binding EF-hand family protein	Actin or actin-binding cytoskeletal protein	-	Calcium ion binding
At3g08720	*ATPK2/ATPK19/S6K2*	Serine/Threonine protein kinase 2	Protein modifying enzyme	Cellular response to hypoxia, response to heat	Protein serine/threonine kinase activity
At3g13790	*CELL WALL INVERTASE1 CWINV1*	Glycosyl hydrolases family 32 protein	-	Defense response to fungus, response to wounding	Hydrolyzing O-glycosyl compounds
At3g15210	*ERF4*	Ethylene responsive element binding factor 4	DNA-binding transcription factor	Induced systemic resistance, cellular response to hypoxia	DNA-binding transcription factor activity
At3g19580	*AZF2*	C2H2-type zinc-finger protein 2	C2H2 zinc finger transcription factor	Response to stress, response to abscisic acid	DNA-binding transcription factor activity
At3g30180	*BR6OX2 /CYP85A2*	Brassinosteroid-6-oxidase 2/ cytochrome P450 85A2	Oxygenase	Brassinosteroid biosynthetic process, oxidation-reduction process	Monooxygenase activity
At3g46620	*RDUF1*	Zinc finger (C3HC4-type RING finger) family protein	Ubiquitin-protein ligase	Response to chitin, response to abscisic acid	Ubiquitin protein ligase activity
At3g54730	At3g54730	Transcription repressor	-	Negative regulation of transcription, DNA-templated	-
At3g55950	*CCR3*	CRINKLY4 related 3	Non-receptor serine/threonine protein kinase	Protein phosphorylation	Kinase activity
At3g56710	*SIB1*	Sigma factor binding protein 1	-	Defense response to bacterium, incompatible interaction	Protein binding
At3g59930	At3g59930	Defensin-like protein	-	-	-
At4g01250	*WRKY22*	WRKY family transcription factor	Transcription factor	Response to chitin, cellular response to hypoxia	DNA-binding transcription factor activity
At4g02540^1^	At4g02540	Cysteine/Histidine-rich C1 domain family protein	-	-	-
At4g20860	*BBE22/CELLOX*	FAD-binding Berberine family protein	-	Positive regulation of H_2_O_2_ biosynthetic process, response to jasmonic acid	FAD binding
At4g24380	At4g24380	Dihydrofolate reductase	Esterase	-	-
At4g25390	At4g25390	Protein kinase superfamily protein	-	Protein phosphorylation	Protein serine/threonine kinase activity
At4g29740	*CKX4*	Cytokinin oxidase 4	Oxidase	Cytokinin metabolic process, oxidation-reduction process	Oxidoreductase activity
At4g30280	*XTH18*	Xyloglucan endotransglucosylase/hydrolase 18	Hydrolase	Cellular response to hypoxia, ell wall biogenesis	Xyloglucan:xyloglucosyl transferase activity
At4g34000^1^	*ABF3*	Abscisic acid responsive elements-binding factor 3	Basic leucine zipper transcription factor	Response to water deprivation, response to abscisic acid	DNA-binding transcription factor activity
At4g34410	*ERF109/RRTF1*	Ethylene responsive element binding factor 109/Redox responsive transcription factor 1	DNA-binding transcription factor	Defense response to fungus, root regeneration	DNA-binding transcription factor activity
At4g37260	*MYB73*	MYB domain protein 73	Transcription factor	Response to chitin, glucosinolate metabolic process	DNA-binding transcription factor activity
At5g03210	*DIP2*	DNA-binding protein phosphatases (DBP)-interacting protein 2	E3 ubiquitin-protein ligase	Defense response to virus	-
At5g05410	*DREB2A*	Dehydration-responsive element binding protein 2A	Transcription factor	Response to stress	DNA-binding transcription factor activity
At5g65070	*MAF4*	MADS AFFECTING FLOWERING 4/K-box region and MADS-box transcription factor family protein	Transcription factor	Negative regulation of flowering development	Transcription regulatory region sequence-specific DNA binding
At5g65080	*MAF5*	MADS AFFECTING FLOWERING 5/K-box region and MADS-box transcription factor family protein	Transcription factor	Negative regulation of flowering development	DNA-binding transcription factor activity

*^1^*genes whose transcript levels were lower in *ldl1 ldl2* than WT plants.**

To analyze the functional relationships among these 39 DEGs, we carried out STRING analysis^3^ (Szklarczyk et al., 2019). The primary biological process of 23 of the 39 genes was ‘response to stimulus’ [*p* = 6.07e-07, false discovery rate (FDR)], and among these genes, 8 were involved in ‘response to chitin and bacterium’ (*p* = 4.55e-09, FDR) (Supplementary Figure 5E). Eleven genes, including 4 AP2 domain-containing transcription factor genes (*ERF4*, *ERF22*, *ERF109*/*RRTF1*, and *DREB2A*) and 3 WRKY transcription factor genes (*WRKY22*, *WRKY25*, and *WRKY40*), were misregulated in the *ldl1 ldl2* mutants at the transcriptional level (*p* = 4.55e-09, FDR) (Supplementary Figure 5E). In the small network composed of the 39 DEGs generated by STRING analysis, WRKY40 might act as a core protein, as it is co-expressed with 13 genes (Supplementary Figure 6A). Additionally, even outliers in this network such as *At1g72910*, *At1g77960*, *CCR3*, and *CKX4* were also differentially transcribed in plants with enhanced disease resistance or whose corresponding mutants were susceptible to *Pseudomonas* infection [Table 1 and Supplementary Figure 6A (gray dot)] (Adams-Phillips et al., 2008; Thatcher et al., 2015; Mendy et al., 2017; Nasim et al., 2020). We also performed STRING network analysis to examine if these *WRKY*s and *ERF*s were co-expressed with some of the defense-related genes tested in this study. Most genes were co-expressed with *WRKY40* and *WRKY70* in the small network (Supplementary Figure 6B). Thus, we propose that the rapid and robust expression of defense-related genes in *ldl1 ldl2* is due to the upregulation of these genes, *e.g*., *WRKY22*, *WRKY40*, and *WRKY70*, and that these 39 DEGs play major roles in the enhanced immunity of the *ldl1 ldl2* mutants.

### LDL1 and LDL1 Are Responsible for the Maintenance of Monomethylated Histone H3K4

**FIGURE 5:**
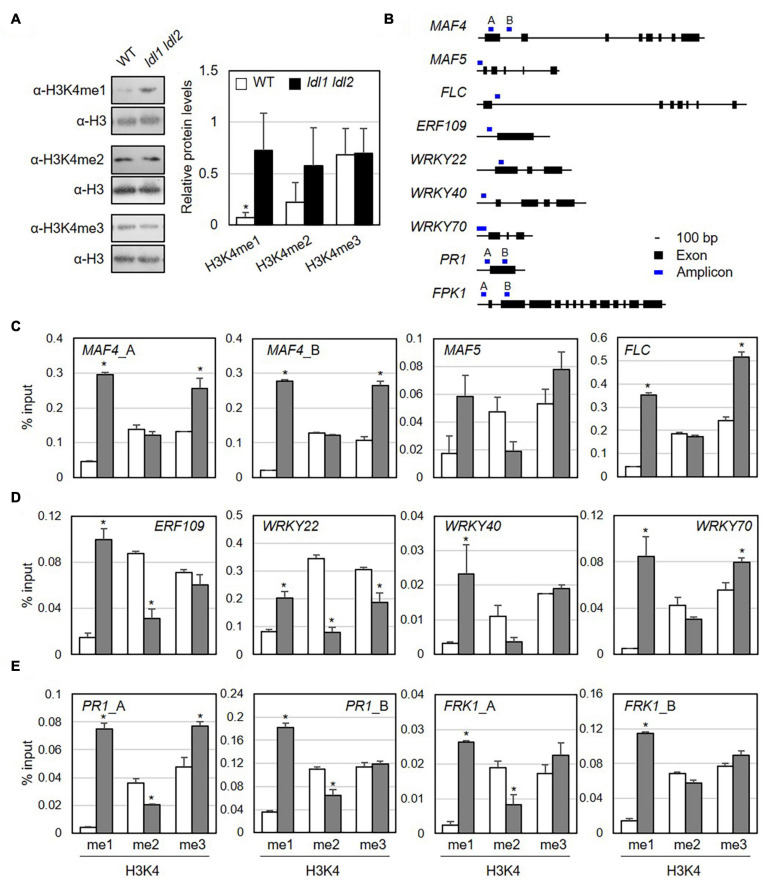
Monomethylation of histone H3K4 is associated with the transcription of genes encoding transcription factors in *Arabidopsis*. **(A)** Abundance of H3K4me1, H3K4me2, and H3K4me3 marked proteins in WT and *ldl1 ldl2* plants. The right panel shows modified histone H3 protein levels in *Arabidopsis* (average ± SD, *p < 0.05, Student’s *t*-test, *n* = 3). The results of all independent experiments are shown in Supplementary Figure 6. **(B)** Genomic DNA regions of gene-specific primers used in panels **(C-E)**. **(C-E)** ChIP-qPCR to examine the enrichment of modified histone H3 proteins in the identified LDL1-target genes **(C)**, transcription factors overrepresented in the *ldl1 ldl2* mutants **(D)**, and *PR1* and *FRK1* genes **(E)**. Enrichment of Lys 4- mono-, Lys 4- di-, and Lys 4-trimethylated histone H3 protein at the chromatin regions of genes was measured in wild type (white bars) and *ldl1 ldl2* (gray bars). The amount of DNA after ChIP was quantified by qPCR, and the means represent the average immunoprecipitation efficiencies (%) against total input DNA used. Each plot shows a representative of two independent biological replications (with similar results), and the data points are the average values of technical triplicates (average ± SEM, *p < 0.05, Student’s *t*-test, *n* = 3). The blue bars are the amplicon regions used for ChiP-qPCR, and the primer sequences used in this study are shown in Supplementary Table 2.

Plant LDL proteins remove methyl groups from histone H3K4 (Hung et al., 2018, 2019). To examine which specific methylation modifications were significantly altered by the simultaneous mutation of *LDL1* and *LDL2* in the absence of pathogen infection, we isolated nuclei from WT and *ldl1 ldl2* plants and performed immunoblot analysis with α-H3K4me1, α-H3K4me2, and α-H3K4me3 antibodies to analyze the protein abundance of modified histone H3 proteins. Lys-4 monomethylated histone H3 (H3K4me1) proteins were present at significantly higher levels in the *ldl1 ldl2* mutants compared to WT plants (Figure 5A and Supplementary Figure 7). The levels of histone H3K4me2 proteins also appeared to be higher in *ldl1 ldl2* than WT plants in two out of three independent experiments, although these differences were not significant (Figure 5A and Supplementary Figure 7B). By contrast, no differences in the levels of histone H3K4me3 proteins were detected between WT and *ldl1 ldl2* (Figure 5A and Supplementary Figure 7C). These observations support the notion that *Arabidopsis* LDL1 and LDL2 preferentially regulate H3K4 monomethylation, as previously described (Hung et al., 2018, 2019).

To determine if specific modifications occur in loci harboring putative target genes, we examined the enrichment of modified histone H3 proteins in their chromatin regions by performing ChIP-qPCR using α-H3K4me1, α-H3K4me2, and α-H3K4me3 antibodies (Figure 5B and Table 1). The promoter and first exon and intron regions of the MADS-box genes *MAF4* and *MAF5/AGL68* and *FLC* are subject to LDL4/FLD-mediated modification, as the trimethylated histone (H3K4me3) levels in these DNA regions were higher in the *fld* mutant vs. the WT (Yu et al., 2011). Furthermore, LDL1 and LDL2 redundantly repress *FLC* expression via H3K4 demethylation (Jiang et al., 2007).

Next, we measured the accumulation of Lys-4 methylated histone H3 proteins on the *MAF4*, *MAF5*, and *FLC* DNA regions as positive controls to verify our experimental procedure. The levels of H3K4me1 and H3K4me3 proteins were higher at *MAF4*, *MAF5*, and *FLC* chromatin in *ldl1 ldl2* vs. WT plants, supporting the previous observation that LDL1 and LDL2 function as histone demethylases (Figure 5C and Supplementary Figure 8) (Jiang et al., 2007; Hung et al., 2018). Hence, we analyzed the level of methylated H3K4 on a few putative targets of LDL1 and LDL2, including *ETHYLENE RESPONSIVE ELEMENT BINDING FACTOR109*/*REDOX RESPONSIVE TRANSCRIPTION FACTOR1* (*ERF109*/*RRTF1*) and the WRKY transcription factors genes *WRKY22*, *WRKY40*, and *WRKY70* (Figure 5D and Supplementary Figure 8). Indeed, more Lys-4 methylated histone H3, especially H3K4me1, accumulated on the promoter regions of these genes in *ldl1 ldl2* vs. WT plants (Figure 5D and Supplementary Table 3).

Finally, because the *ldl* mutants exhibited higher disease resistance due to rapid and higher expression of *PR1* (Figure 2B) and *FRK1* (Figure 3) vs. the WT, we examined whether LDL1 and LDL2 are involved in histone methylation of the promoter and coding regions of these genes. The level of H3K4me1 increased in *ldl1 ldl2* on all amplicons of *PR1* and *FRK1*. Interestingly, the expression of *PR1* and *FRK1* before bacterial infection (0 hpi) in the *ldl* single and double mutants was not significantly different from that of WT plants, likely because LDL1 and LDL2 act synergistically with histone deacetylase complex (HDAC) to repress gene expression (Hung et al., 2018, 2019, 2020). These results indicate that the H3K4me1 state in several defense-related genes primes plants to respond to subsequent *Pseudomonas* infection in a sensitive manner.

## Discussion

Our findings suggest that the *Arabidopsis* histone demethylase proteins LDL1 and LDL2 are required to maintain the switched-off state of the immune response under uninfected conditions. The individual and simultaneous mutations of both genes render *Arabidopsis* plants resistant to virulent *Pseudomonas* infection via the increased expression of SA- and MAMP-responsive genes after infection. These LDL1 and LDL2 proteins are key players that detach the monomethyl group from histone H3K4 proteins that occupy defense-related genes, such as *WRKY*s, *ERF*s, *PR1*, and *FRK1*. Thus, we hypothesized that an epigenetic eraser(s) associated with LDL1 and LDL2, which removes the methyl groups at histone H3K4 residues in the chromatin regions of defense-related genes, is vital for the primed defense response in *Arabidopsis*.

Unlike other histone methylation marks (H3K9, H3K27, and H4K20), histone H3K4, H3K36, and H3K79 methylation are euchromatic histone modifications (Black et al., 2012). Changes in H3K4 methylation patterns influence the immune responses of *Arabidopsis* and rice plants (Lee et al., 2016; Ramirez-Prado et al., 2018). The mutation of *Arabidopsis TRITHORAX1*/*SET DOMAIN GROUP27* (*ATX1/SDG27*), encoding a H3K4 methyltransferase, led to reduced H3K4me2 and H3K4me3 levels. The *atx1* mutants, which exhibit low *PR1* and *WRKY70* transcript levels, are susceptible to *Pst*DC3000 infection (Alvarez-Venegas et al., 2003, 2007; Alvarez-Venegas, 2005). *ATX-RELATED7*/*SET DOMAIN GROUP 25* (*ATX7*/*SDG25*) also plays a role in H3K4 methylation. The *atx7*/*sdg25* mutants, with impaired monomethylation of H3K4, show increased susceptibility to infection by *Pst*DC3000 and *Botrytis cinerea* (Lee et al., 2016). The demethylase JMJ704 positively regulates the immunity response of rice by suppressing the expression of genes encoding negative regulators of defense responses (Hou et al., 2015). By contrast, we demonstrated that *ldl1 ldl2* double mutants showed increased H3K4me1 accumulation at the whole chromatin level and were enriched for H3K4me1 at the defense-related genes examined in this study. Together, these findings demonstrate that methylation of histone H3K4 is vital for the expression of defense-related genes and disease resistance responses during infection.

Since LDLs erase histone H3K4 methylation marks, especially mono-methylation, we cautiously suggest that LDL1 and LDL2 function as counterparts of ATX1/SDG27 and ATX7/SDG25 to fine-tune the methylation levels of histone H3K4 at the chromatin of defense-related genes in *Arabidopsis*. SAR-defective *ldl4*/*fld*/*rsi1* mutants displayed reduced H3K4me2 levels at the *WRKY6* and *WRKY29* loci, which encode positive regulators of the immune response (Singh et al., 2013; Singh V. et al., 2014). Therefore, *Arabidopsis* LDLs may fine-tune plant immune responses in a sophisticated manner by targeting different genes. Indeed, *ldl1*, *ldl2*, and *ldl4*/*fld* exhibit a late-flowering phenotype, although the tissue- and organ-specific expression patterns of these genes and the targets of the encoded proteins are different (Greenberg et al., 2013; Yu et al., 2016; Hung et al., 2018; Martignago et al., 2019). For example, 2.3% of DEGs (60 genes out of 2539 DEGs) and 5.8% of DEGs (17 genes out of 273) appear to be involved in biotic stress responses in *ldl4*/*fld* and *ldl1 ldl2*, respectively (Yu et al., 2016; Supplementary Figure 5A). In addition, among *WRKY* genes (*WRKY22*, *25*, *33*, *40*, *26*, *48*, and *53*) whose expression increased in the *ldl1 ldl2* double mutants, the expression of four *WRKY*s (*WRKY22*, *33*, *40*, and *53*) was reduced in the *ldl4*/*fld* mutants (Yu et al., 2016; Supplementary Table 3). These findings suggest that all LDL histone demethylases function as epigenetic erasers but target different genes in the immune response.

Among the four homologous LDL group members, LDL1 and LDL2 are highly similar to LDL4/FLD structurally, with an N-terminal SWIRM domain and an amine oxidase domain, while the structure of LDL3 is different from the others (Martignago et al., 2019). Like the *ldl4*/*fld* mutant, mutations of *LDL1* and *LDL2* result in late flowering due to high expression of *FLC* and *FWA*, but the *ldl3* mutants flower earlier and express *FLC* at lower levels than WT plants (Martignago et al., 2019). LDL4/FLD is involved in regulating flowering time in cooperation with the histone deacetylase HDA6 by controlling the occupation of acetylated and methylated histone proteins (H3K9K14Ac and H3K4Me3) on the DNA regions of *FLC*, *MAF4*, and *MAF5*, encoding floral repressors (Yu et al., 2011). LDL1/LDL2 also form a complex with HDA6, which functions as a negative transcriptional regulator of its target genes by switching its interacting partners. For example, the HDA6-LDL1/LDL2 complex associates with transcription factors such as CIRCADIAN CLOCK ASSOCIATED 1 (CCA1), LATE ELONGATED HYPOCOTYL (LHY), and TIMING OF CAB EXPRESSION 1 (TOC1), which function as key circadian clock oscillators in a nested feedback loop to maintain circadian rhythms (Hung et al., 2018, 2019). The occupation of acetylated and/or methylated histone 3 proteins is significantly higher in *hda6* and *ldl* mutants compared to WT. H3Ac and H3K4me level are likely higher in *ldl1 ldl2* than the WT. Furthermore, the levels of H3Ac and H3K4me2 are higher in *hda6 ldl1 ldl2* than in *hda6* and *ldl1 ldl2*. In addition, *TOC1* transcript levels are much higher in *hda6 ldl1 ldl2* than in the single mutants (Hung et al., 2018). These findings suggest that histone deacetylase and histone demethylase stabilize the histone modification complex and/or regulate the transcription of their target genes in an additive manner. When we compared the mRNA-seq data generated in the current study to previous RNA-seq data (Yu et al., 2016), even though the plant growth conditions were different, 32 DEGs identified in *ldl1 ldl2* (Supplementary Table 3) were also highly expressed in the *hda6* mutant. Thus, we propose that the transcription and histone modification required for plant immunity are controlled by the HDA6-LDL1/LDL2 complex. The finding that *hda6* and *ldl* mutants are resistant to pathogen infection (Wang et al., 2017 and this study) supports this idea.

The enhanced disease resistance of the *ldl1 ldl2* mutants appears to be due to the upregulation of their putative target genes (Supplementary Figure 6B), including *WRKY*s and *ERF*s. WRKYs are important transcriptional regulators that function in plant defense. *WRKY22* expression is induced during the early stage of bacterial pathogen infection (Dong et al., 2003). Pre-submerged *wrky22* mutants were susceptible to bacterial infection with *Pst*DC3000 compared to WT plants due to the downregulation of its target genes such as *WRKY53* and *FRK1*, which confer innate immunity (Hsu et al., 2013). WRKY25 is involved in various stress responses (heat, salt, oxidative stress, and malnutrition) in addition to the response to bacterial pathogen infection (Zheng et al., 2007; Jiang and Deyholos, 2009; Li et al., 2009; Doll et al., 2020; Wu et al., 2020). WRKY40 negatively regulates PTI and attenuates early defense-induced genes during PTI (Lozano-Durán et al., 2013), but it positively regulates ETI via the Toll/Interleukin 1 Receptor (TIR)-type NLR RPS4 (Schön et al., 2013), whereas *wrky70* mutants exhibited upregulated expression of disease-responsive genes such as *PR1* and *PDF1.2* (Ülker et al., 2007). Another putative target of LDL1 and LDL2, *ERF109*/*RRTF1* (encoding an ERF/AP transcription factor involved in redox homeostasis), is under the control of WRKY40 (Pandey et al., 2010), and its promoter region appears to be subject to epigenetic changes (Soliman and Meyer, 2019). In addition, ERF109 promotes the expression of S-adenosyl-L-Met-dependent methyltransferase1 (BSMT1), which methylates SA and benzoic acid (Lin et al., 2020) and is thought to mediate crosstalk between jasmonic acid and auxin signaling to regulate lateral root formation (Cai et al., 2014) and various biotic and abiotic stress responses (Bahieldin et al., 2018). Thus, the altered expression of several *WRKY*s and *ERF*s, which are targets of LDL1 and LDL2, can explain the disease phenotype of the *ldl* mutants.

Notably, unlike the *ldl4*/*fld*/*rsi1* mutants, which are defective in SAR but show WT-like local resistance against both virulent and avirulent *Pseudomonas* strains (Singh et al., 2013), loss-of-function mutations in *LDL1* and *LDL2* resulted in disease resistance against virulent bacterial infection along with the upregulated expression of defense-related genes without any increase in SA levels or callose deposition after pathogen infection (Figures 1–3 and Supplementary Figures 2, 3). Unlike well-characterized mutants with enhanced disease resistance, in which SA signaling and defense gene expression are constitutively active (Lorrain et al., 2003), analysis of *ldl1* and *ldl2* mutants indicated that LDL1 and LDL2 do not appear to directly control SA- and MAMP-dependent defense responses. The current observations point to the possibility that the *ldl1* and *ldl2* mutants might be in a defense priming state in the absence of any stimuli. Systemic resistance also results from defense priming, rendering plants sensitive to subsequent external stimuli, a process involving chromatin modification (Bruce et al., 2007; Conrath et al., 2015; Ding and Wang, 2015). Local immunization with SAR-inducing *P. syringae* pv. *maculicola* induced several covalent modifications of histone proteins, such as H3K4me2, H3K4me3, H4K5ac, H4K8ac, and H4K12ac, at the promoter regions of *WRKY6*, *WRKY29*, and *WRKY53* in systemic leaves (Jaskiewicz et al., 2011). *WRKY29* transcript levels, however, were not altered in the systemic leaves of immunized plants prior to secondary challenge-inoculation. Similar histone modifications and gene expression patterns were observed in benzothiadiazole-treated leaves before and after exposure to a secondary stimulus (Jaskiewicz et al., 2011). In line with these results, the transcription of defense-related genes increased significantly in local leaves of *ldl1*, *ldl2*, and *ldl1 ldl2* compared to WT plants after pathogen infection (Figures 2–4 and Supplementary Figures 2–6). Furthermore, monomethylated histone H3K4 proteins predominantly occupied the promoter regions of defense-related genes (Figure 5 and Supplementary Figure 8). Our findings support the idea that histone modification, especially methylation of histone H3K4, reconstructs a docking region on chromatin for specific transcriptional activators, which would be rapidly activated upon subsequent stimuli (de la Cruz et al., 2005; Vermeulen et al., 2007; Ding et al., 2012). Considering the intensified transcription that occurs after local pathogen infection, it seems reasonable that transcriptional activation could occur in the systemic leaves of mock-immunized mutant plants. Taken together, these findings suggest that histone eraser complexes harboring LDL1 and LDL2 are involved in the proper growth/development and immunity responses of plants, likely in conjunction with various transcription factors (Supplementary Figure 9).

## Data Availability Statement

The original contributions presented in the study are publicly available. This data can be found here: NCBI repository, Accession Number: GSE171433.

## Author Contributions

SWN, R-RS, and HJP performed the experiments. HJP and HWJ analyzed the data and wrote the manuscript. HWJ designed the study. All authors contributed to the article and approved the submitted version.

## Conflict of Interest

The authors declare that the research was conducted in the absence of any commercial or financial relationships that could be construed as a potential conflict of interest.

## Footnotes

1 http://nabic.rda.go.kr/

2 http://www.bar.utoronto.ca/

3 https://string-db.org

## Supplementary Material

Supplementary Figure 1 Mutations of two different *LDL* genes render *Arabidopsis* plants resistant against virulent *Pseudomonas* infection. **(A)** Transcriptional changes in *Arabidopsis LDL* genes in local leaves of WT plants during pathogenesis. The expression levels of four *Arabidopsis LDL* homologs in local leaves upon bacterial infection were examined using eFP visualized meta-analysis (http://www.bar.utoronto.ca/) (Winter et al., 2007). Input data were obtained from microarray data set using ATH1 GeneChip showing mRNA levels in leaves of 4-week-old WT plants inoculated with 10 mM MgSO_4_ as the mock treatment (M), *P. syringae* pv. *maculicola* ES4326 carrying *AvrRpt2* (*Psm*ES4326/*AvrRpt2*) as the avirulent pathogen (A), and *Psm*ES4326 as the virulent pathogen (V). **(B)** Scheme for pathogen inoculation and sampling times of plant tissue in panel **(C)**. Leaves of 4-week-old WT plants were pre-immunized with *Psm*ES4326 carrying *AvrRpt2* (*Psm*ES4326/*AvrRpt2*, DG6) (OD_600_ = 0.01, F), and 2 days later, distal leaves were infiltrated with *Psm*ES4326 (OD_600_ = 0.01, S) using a needleless syringe. **(C)** RT-qPCR to measure the transcript levels of *LDL* homologous genes in non-infected local leaves (F) and distal leaves of immunized plants (S) at 0, 10, and 20 h post-inoculation (hpi). *ACTIN2* was used to normalize the expression of the *LDLs*. Relative expression is shown as the average ± SD. Gray arrows indicate significant differences from non-infected local leaves (*p* > 0.05, two-tailed Student’s *t*-test, *n* = 11, each biological replicate contained three technical repeats). **(D)** *Pseudomonas* growth in the leaves of WT, *ldl1*, *ldl2*, *ldl3*, and *ldl4*/*fld* plants after *Psm*ES4326 infection. **(E)** Enhanced resistance of different alleles of *ldl1* and *ldl2* mutants to virulent *Pseudomonas* infection. Virulent *Psm*ES4326 **(D,E)** and *P. syringae* pv. *tomato* DC3000 (*Pst*DC3000) **(E)** strains (OD_600_ = 0.0001) were used in these studies. Bacterial titers were evaluated at 3 dpi. The area of a leaf disc is 0.78 cm^2^. Averages ± SEM are plotted, and asterisks indicate significant differences from WT plants, as determined by a two-tailed Student’s *t*-test (^∗^*p* < 0.05, ^∗∗^*p* < 0.01, *n* = 8). The experiments were repeated over 3 times with similar results. **(F)** List of tested T-DNA insertion mutants corresponding to each LDL gene in *Arabidopsis*.

Supplementary Figure 2 Transcript levels of SA-responsive genes in local leaves of WT and mutant plants upon infection with virulent strain *Psm*ES4326 (OD_600_ = 0.01). **(A**,**B)** Each plot is representative of the five biological replicates of *PR1* **(A)**, and *NPR1* **(B)** shown in Figure 2B. **(C,D)** Transcript levels of *ALD1* **(C)** and *WRKY70* **(D)** in *Arabidopsis* leaves during infection. Tables below each plot indicate the number of independent trials with significant differences between WT and a given plant genotype. The symbols represent the following: ↑, mRNA levels were upregulated in the mutants; =, mRNA levels in the mutants were comparable to those of WT plants; ↓, mRNA levels were downregulated in the mutants. The averages ± SD (standard deviation) were plotted (^∗^*p* < 0.05, ^∗∗^*p* < 0.01, two-tailed Student’s *t*-test, *n* = 3).

Supplementary Figure 3 Transcript levels of MAMP-responsive genes in local leaves of WT and mutant plants upon infection with virulent strain *Psm*ES4326 (OD_600_ = 0.01). **(A**-**C)** Each plot is representative of one of the four independent biological replicates of *FRK1* **(A)**, *NHL10* **(B)**, and *At1g51890* **(C)** shown in Figure 3C. Bar indicates the SD (^∗^*p* < 0.05, ^∗∗^*p* < 0.01, two-tailed Student’s *t*-test, *n* = 3).

Supplementary Figure 4 Transcript levels of defense-related genes in distal systemic leaves of WT and mutant plants upon challenge-inoculation with virulent *Pseudomonas*. **(A,B)** Each plot represents the other biological repeat of *PR1* **(A)**, and *FRK1* **(B)** in Figures 4C,D. **(C,D)** Transcript levels of *NPR1* **(C)** and *WRKY70* **(D)** in distal leaves during SAR. Bar indicates the SD (^∗^*p* < 0.05, ^∗∗^*p* < 0.01, two-tailed Student’s *t*-test, *n* = 3).

Supplementary Figure 5 GO analysis of DEGs in *ldl1 ldl2* mutants and identification of LDL1- and LDL2-target genes that function in plant immunity. **(A**-**C)** Gene Ontology (GO) enrichment by the Panther classification system (Szklarczyk et al., 2019) of 273 DEGs in the biological process **(A)**, molecular function **(B)**, and protein class **(C)** categories in *ldl1 ldl2* compared with WT. Annotated terms with *p* < 1.E-04 **(A,B)** and *p* < 2.51E-03 **(C)** are listed. (**D**) Venn diagram of DEGs in *ldl1 ldl2* and LDL1-target genes. DEGs identified by microarray analysis of *ldl1 ldl2* mutants (Berr et al., 2015) and using ChIP-seq data obtained from LDL1-GFP expressing plants (Hung et al., 2018) were compared with DEGs in the *ldl1 ldl2* mutant identified by RNA-seq analysis. Numbers in parentheses are the total numbers of genes identified in each study. **(E)** 23, 11, and 8 genes involved in ‘response to stimulus’ (red dot), ‘DNA-binding transcription factor activity’ (blue dot), and ‘response to chitin and to bacterium’ (green dot), respectively, and their False Discovery Rates (FDR) are shown in the table.

Supplementary Figure 6 STRING analysis of 39 DEGs and representative defense-related genes. **(A)** Thirty-nine proteins that are putative targets of LDLs (Table 1) were subjected to protein-protein interaction network functional enrichment analysis via STRING (Mi et al., 2013). Dots represent the putative functions of these proteins, including ‘response to stimulus’ (red dots), ‘DNA-binding transcription factor activity’ (blue dots), and ‘response to chitin and to bacterium’ (green dots) (Supplementary Figure 5E) **(B)** Proteins involved in SA signaling pathways (PR1, NPR1, and ALD1), the PTI pathway (WRKY70, WRKY25, RFK1, YLS9, and AT1G51890), and a few putative targets of LDLS (WRKY22, WRKY40, ERF1, and RRTF1) were subjected to STRING analysis.

Supplementary Figure 7 Levels of modified histone H3 protein in WT and *ldl1 ldl2*. **(A**-**C)** Nuclear proteins extracted from three independent biological replications (1^*o*^, 2^*o*^, and 3^*o*^) were subjected to SDS-PAGE, and immunoblot analysis was performed using α-H3K4me1 **(A)**, α-H3K4me2 **(B)**, and α-H3K4me3 **(C)** antibodies. Numbers indicate independent experimental trials. w, WT plants; m, *ldl1 ldl2*. The normalized and processed data were plotted in Figure 5A.

Supplementary Figure 8 Monomethylation of histone H3K4 is associated with the transcription of genes encoding transcription factors in *Arabidopsis.* The plots represent the second of two independent biological replications of ChIP-PCR to examine the enrichment of modified histone H3 proteins in the identified LDL1-target genes **(A)**, transcription factors overrepresented in *ldl1 ldl2* mutants **(B)**, and *FRK1* genes **(C)**.

Supplementary Figure 9 A proposed model about the role of LDL1 and LDL2 in the transcription of defense-related genes in *Arabidopsis*. A previous study reveals that *Arabidopsis* requires ATX1/SDG25 for monomethylation of histone H3K4, and its KO mutants were susceptible to *Pst*DC3000 infection. On another side, both LDL1 and LDL2 are responsible for suppressing the immoderate expression of defense-related genes in the absence of pathogen infection. Thus, it is likely that the prevention of detaching methyl group of H3K4 in nucleosome associated with defense-related genes is enough to prime plants against subsequent pathogen infection.

Supplementary Table 1 Bacterial strains used in this study.

Supplementary Table 2 Oligonucleotide sequences used in this study.

Supplementary Table 3 DEGs in the *ldl1 ldl2* mutants, including DEGs that overlap with LDL1-target genes.
