# Supplementary material for: Two Arabidopsis Homologs of Human Lysine-Specific Demethylase Function in Epigenetic Regulation of Plant Defense Responses
Source: Front Plant Sci. 2021 Jun 14;12:688003. doi: 10.3389/fpls.2021.688003 (PMC8236864; doi:10.3389/fpls.2021.688003)
Supplement: Supplementary Figure 1 — Mutations of two different LDL genes render Arabidopsis plants resistant against virulent Pseudomonas infection. (A) Transcriptional changes in Arabidopsis LDL genes in local leaves of WT plants during pathogenesis. The expression levels of four Arabidopsis LDL homologs in local leaves upon bacterial infection were examined using eFP visualized meta-analysis (http://www.bar.utoronto.ca/) (Winter et al., 2007). Input data were obtained from microarray data set using ATH1 GeneChip showing mRNA levels in leaves of 4-week-old WT plants inoculated with 10 mM MgSO4 as the mock treatment (M), P. syringae pv. maculicola ES4326 carrying AvrRpt2 (PsmES4326/AvrRpt2) as the avirulent pathogen (A), and PsmES4326 as the virulent pathogen (V). (B) Scheme for pathogen inoculation and sampling times of plant tissue in panel (C). Leaves of 4-week-old WT plants were pre-immunized with PsmES4326 carrying AvrRpt2 (PsmES4326/AvrRpt2, DG6) (OD600 = 0.01, F), and 2 days later, distal leaves were infiltrated with PsmES4326 (OD600 = 0.01, S) using a needleless syringe. (C) RT-qPCR to measure the transcript levels of LDL homologous genes in non-infected local leaves (F) and distal leaves of immunized plants (S) at 0, 10, and 20 h post-inoculation (hpi). ACTIN2 was used to normalize the expression of the LDLs. Relative expression is shown as the average ± SD. Gray arrows indicate significant differences from non-infected local leaves (p > 0.05, two-tailed Student’s t-test, n = 11, each biological replicate contained three technical repeats). (D) Pseudomonas growth in the leaves of WT, ldl1, ldl2, ldl3, and ldl4/fld plants after PsmES4326 infection. (E) Enhanced resistance of different alleles of ldl1 and ldl2 mutants to virulent Pseudomonas infection. Virulent PsmES4326 (D,E) and P. syringae pv. tomato DC3000 (PstDC3000) (E) strains (OD600 = 0.0001) were used in these studies. Bacterial titers were evaluated at 3 dpi. The area of a leaf disc is 0.78 cm2. Averages ± SEM are plotted, and asteris [file Presentation_1.pptx]

## Slide 1
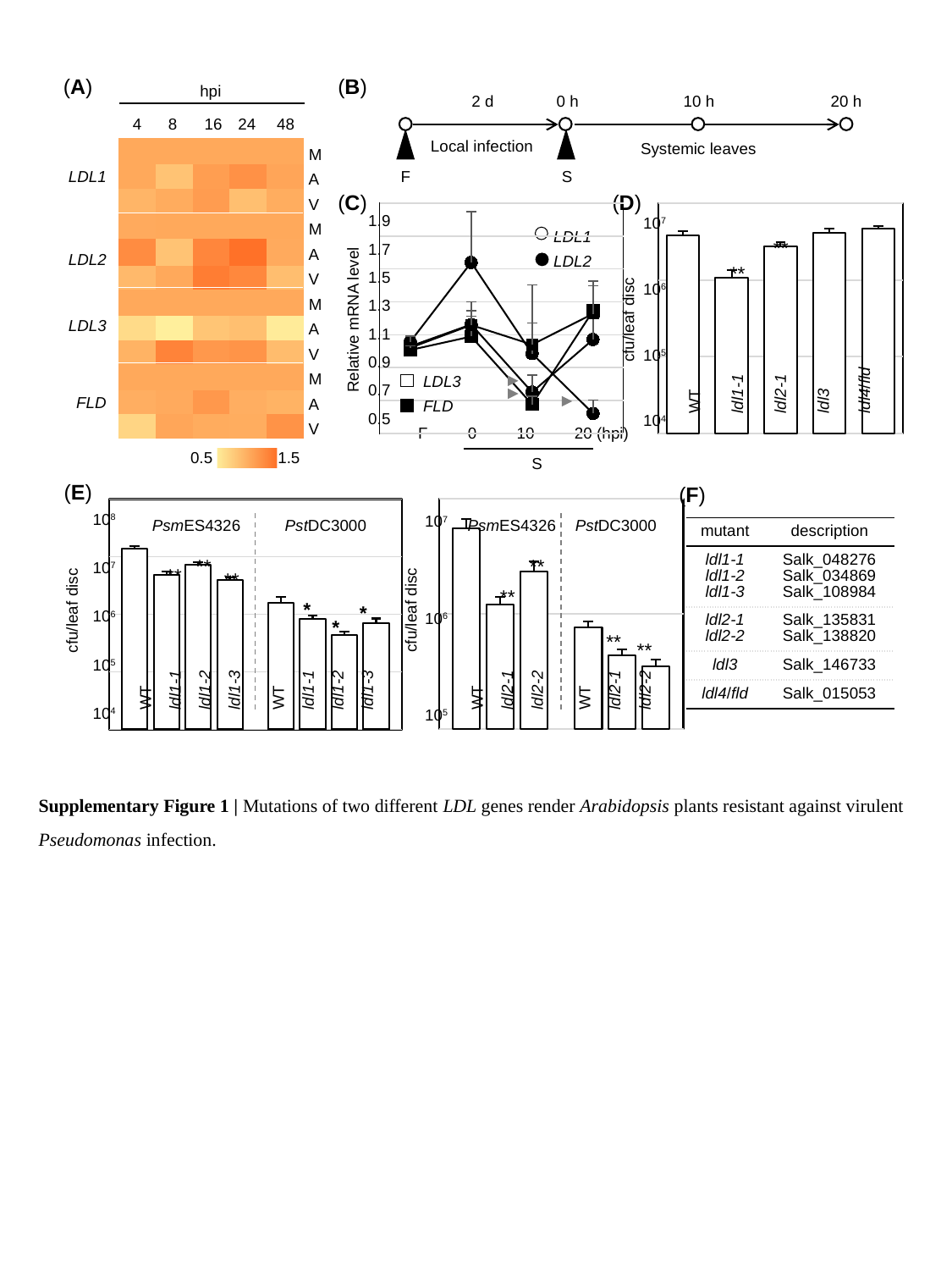

(A)
(B)
hpi
2 d
0 h
10 h
20 h
4
8
16
24
48
Local infection
Systemic leaves
M
F
S
LDL1
A
(C)
(D)
V
### Chart
| Category | | | | |
|---|---|---|---|---|
### Chart
| Category | Pma DG3 |
|---|---|
| Col-0 | 3766666.66666667 |
| eni1 | 1058333.33333333 |
| enl1 | 2708333.333333327 |
| enl2 | 4136363.6363636353 |
| enl3 | 4636363.63636364 |1.9
107
M
LDL1
LDL2
**
1.7
A
LDL2
**
1.5
V
106
M
1.3
Relative mRNA level
cfu/leaf disc
LDL3
A
1.1
V
105
0.9
M
LDL3
FLD
0.7
WT
ldl4/fld
ldl1-1
ldl2-1
ldl3
FLD
A
0.5
104
V
F 0 10 20 (hpi)
0.5
1.5
S
(E)
(F)
### Chart
| Category | Col-0 | enl1 | enl1-2 |
|---|---|---|---|
| Pma DG3 | 5537500.0 | 1199375.0 | 2306875.0 |
| Pst DC3000 | 759375.0 | 436250.0 | 351250.0 |
### Chart
| Category | Col-0 | eni1 | eni1-2 | eni1-3 |
|---|---|---|---|---|
| Pma DG3 | 13687500.0 | 4806250.0 | 7268750.0 | 3987500.0 |
| Pst DC3000 | 1600000.0 | 823125.0 | 431250.0 | 699375.0 |108
107
PsmES4326
PstDC3000
PsmES4326
PstDC3000
**
**
107
**
**
**
*
cfu/leaf disc
*
cfu/leaf disc
106
106
*
**
**
105
WT
WT
WT
WT
ldl1-1
ldl1-2
ldl1-3
ldl1-1
ldl1-2
ldl1-3
ldl2-1
ldl2-2
ldl2-1
ldl2-2
104
105
| | | | | |
| --- | --- | --- | --- | --- |
| | | | | |
| | | | | |
| | | | | |
| | | | | |
| | | | | |
| | | | | |
| | | | | |
| | | | | |
| | | | | |
| | | | | |
| | | | | |
| mutant | description |
| --- | --- |
| ldl1-1 ldl1-2 ldl1-3 | Salk\_048276 Salk\_034869 Salk\_108984 |
| ldl2-1 ldl2-2 | Salk\_135831 Salk\_138820 |
| ldl3 | Salk\_146733 |
| ldl4/fld | Salk\_015053 |
Supplementary Figure 1 | Mutations of two different LDL genes render Arabidopsis plants resistant against virulent Pseudomonas infection.

## Slide 2
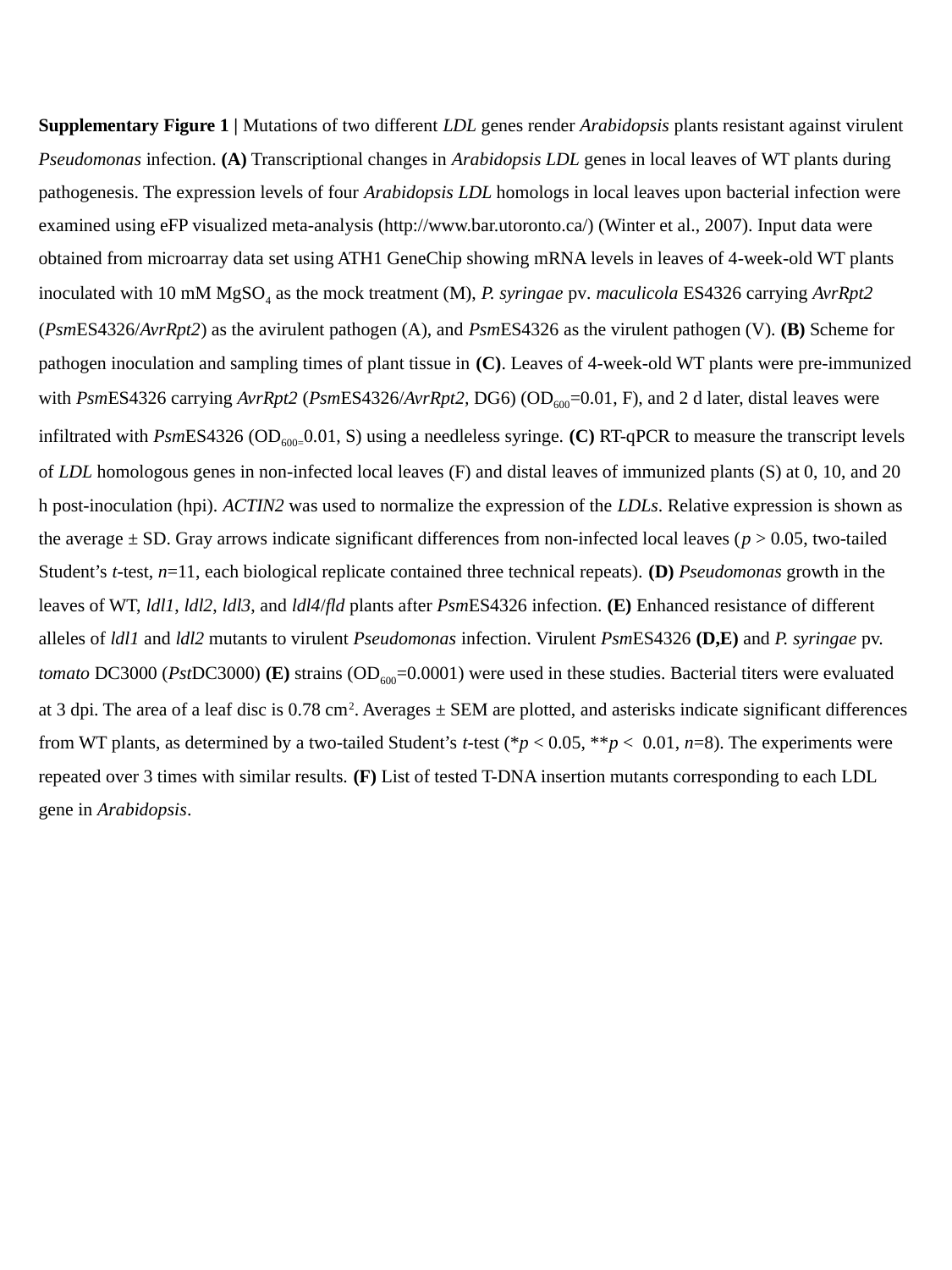

Supplementary Figure 1 | Mutations of two different LDL genes render Arabidopsis plants resistant against virulent Pseudomonas infection. (A) Transcriptional changes in Arabidopsis LDL genes in local leaves of WT plants during pathogenesis. The expression levels of four Arabidopsis LDL homologs in local leaves upon bacterial infection were examined using eFP visualized meta-analysis (http://www.bar.utoronto.ca/) (Winter et al., 2007). Input data were obtained from microarray data set using ATH1 GeneChip showing mRNA levels in leaves of 4-week-old WT plants inoculated with 10 mM MgSO4 as the mock treatment (M), P. syringae pv. maculicola ES4326 carrying AvrRpt2 (PsmES4326/AvrRpt2) as the avirulent pathogen (A), and PsmES4326 as the virulent pathogen (V). (B) Scheme for pathogen inoculation and sampling times of plant tissue in (C). Leaves of 4-week-old WT plants were pre-immunized with PsmES4326 carrying AvrRpt2 (PsmES4326/AvrRpt2, DG6) (OD600=0.01, F), and 2 d later, distal leaves were infiltrated with PsmES4326 (OD600=0.01, S) using a needleless syringe. (C) RT-qPCR to measure the transcript levels of LDL homologous genes in non-infected local leaves (F) and distal leaves of immunized plants (S) at 0, 10, and 20 h post-inoculation (hpi). ACTIN2 was used to normalize the expression of the LDLs. Relative expression is shown as the average ± SD. Gray arrows indicate significant differences from non-infected local leaves (p > 0.05, two-tailed Student’s t-test, n=11, each biological replicate contained three technical repeats). (D) Pseudomonas growth in the leaves of WT, ldl1, ldl2, ldl3, and ldl4/fld plants after PsmES4326 infection. (E) Enhanced resistance of different alleles of ldl1 and ldl2 mutants to virulent Pseudomonas infection. Virulent PsmES4326 (D,E) and P. syringae pv. tomato DC3000 (PstDC3000) (E) strains (OD600=0.0001) were used in these studies. Bacterial titers were evaluated at 3 dpi. The area of a leaf disc is 0.78 cm2. Averages ± SEM are plotted, and asterisks indicate significant differences from WT plants, as determined by a two-tailed Student’s t-test (*p < 0.05, **p < 0.01, n=8). The experiments were repeated over 3 times with similar results. (F) List of tested T-DNA insertion mutants corresponding to each LDL gene in Arabidopsis.

## Slide 3
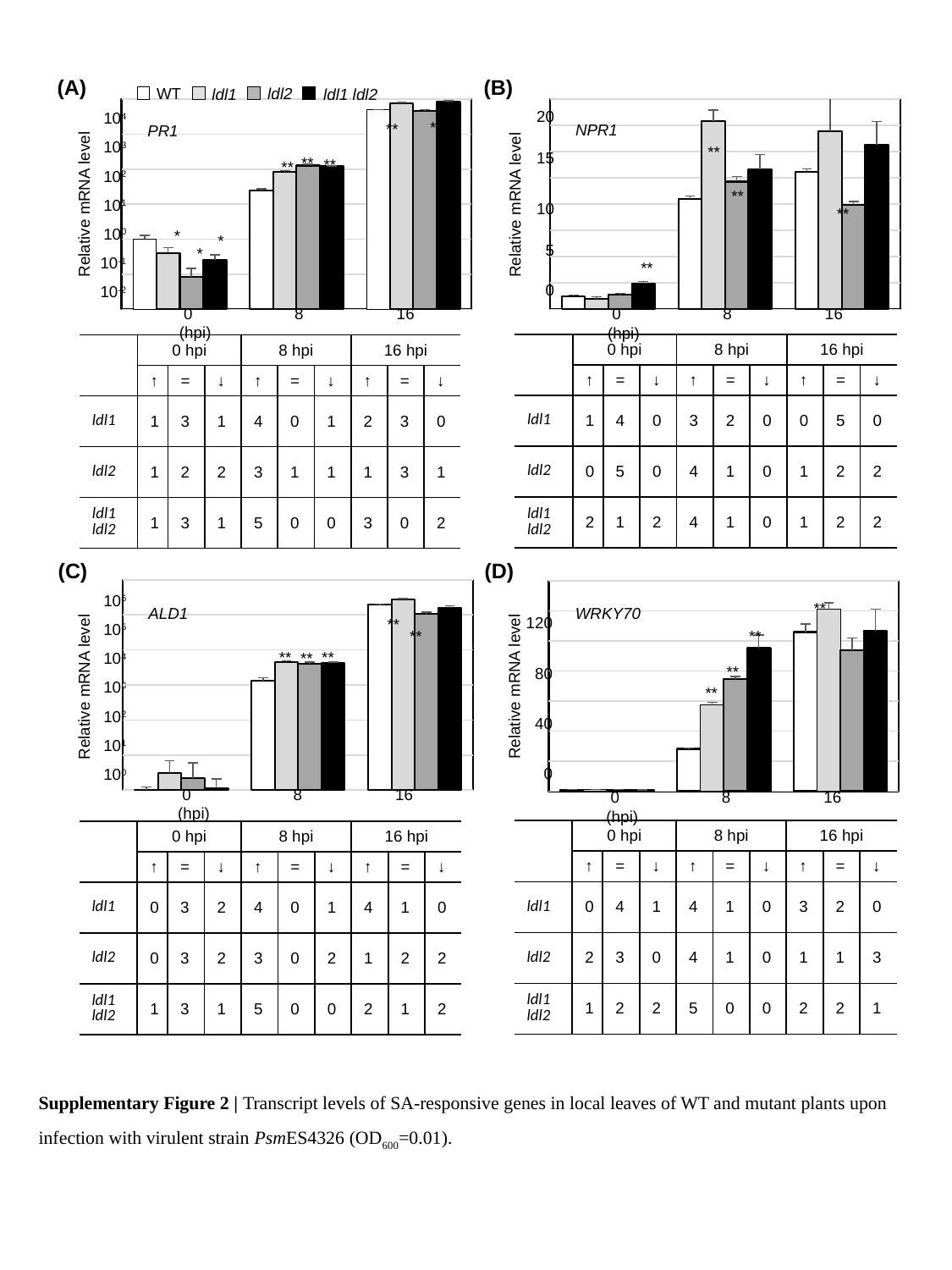

(A)
(B)
WT
ldl2
ldl1
ldl1 ldl2
### Chart
| Category | | | | |
|---|---|---|---|---|104
**
**
PR1
103
**
**
**
102
Relative mRNA level
101
100
*
*
*
10-1
10-2
 0 8 16 (hpi)
### Chart
| Category | | | | |
|---|---|---|---|---|20
NPR1
**
15
*
**
Relative mRNA level
10
**
5
**
0
 0 8 16 (hpi)
(C)
(D)
### Chart
| Category | | | | |
|---|---|---|---|---|106
ALD1
**
105
**
**
**
**
104
Relative mRNA level
103
102
101
100
 0 8 16 (hpi)
### Chart
| Category | | | | |
|---|---|---|---|---|**
WRKY70
120
**
**
80
Relative mRNA level
**
40
0
 0 8 16 (hpi)
| | 0 hpi | | | 8 hpi | | | 16 hpi | | |
| --- | --- | --- | --- | --- | --- | --- | --- | --- | --- |
| | ↑ | = | ↓ | ↑ | = | ↓ | ↑ | = | ↓ |
| ldl1 | 1 | 4 | 0 | 3 | 2 | 0 | 0 | 5 | 0 |
| ldl2 | 0 | 5 | 0 | 4 | 1 | 0 | 1 | 2 | 2 |
| ldl1 ldl2 | 2 | 1 | 2 | 4 | 1 | 0 | 1 | 2 | 2 |
| | 0 hpi | | | 8 hpi | | | 16 hpi | | |
| --- | --- | --- | --- | --- | --- | --- | --- | --- | --- |
| | ↑ | = | ↓ | ↑ | = | ↓ | ↑ | = | ↓ |
| ldl1 | 1 | 3 | 1 | 4 | 0 | 1 | 2 | 3 | 0 |
| ldl2 | 1 | 2 | 2 | 3 | 1 | 1 | 1 | 3 | 1 |
| ldl1 ldl2 | 1 | 3 | 1 | 5 | 0 | 0 | 3 | 0 | 2 |
| | 0 hpi | | | 8 hpi | | | 16 hpi | | |
| --- | --- | --- | --- | --- | --- | --- | --- | --- | --- |
| | ↑ | = | ↓ | ↑ | = | ↓ | ↑ | = | ↓ |
| ldl1 | 0 | 4 | 1 | 4 | 1 | 0 | 3 | 2 | 0 |
| ldl2 | 2 | 3 | 0 | 4 | 1 | 0 | 1 | 1 | 3 |
| ldl1 ldl2 | 1 | 2 | 2 | 5 | 0 | 0 | 2 | 2 | 1 |
| | 0 hpi | | | 8 hpi | | | 16 hpi | | |
| --- | --- | --- | --- | --- | --- | --- | --- | --- | --- |
| | ↑ | = | ↓ | ↑ | = | ↓ | ↑ | = | ↓ |
| ldl1 | 0 | 3 | 2 | 4 | 0 | 1 | 4 | 1 | 0 |
| ldl2 | 0 | 3 | 2 | 3 | 0 | 2 | 1 | 2 | 2 |
| ldl1 ldl2 | 1 | 3 | 1 | 5 | 0 | 0 | 2 | 1 | 2 |
Supplementary Figure 2 | Transcript levels of SA-responsive genes in local leaves of WT and mutant plants upon infection with virulent strain PsmES4326 (OD600=0.01).

## Slide 4
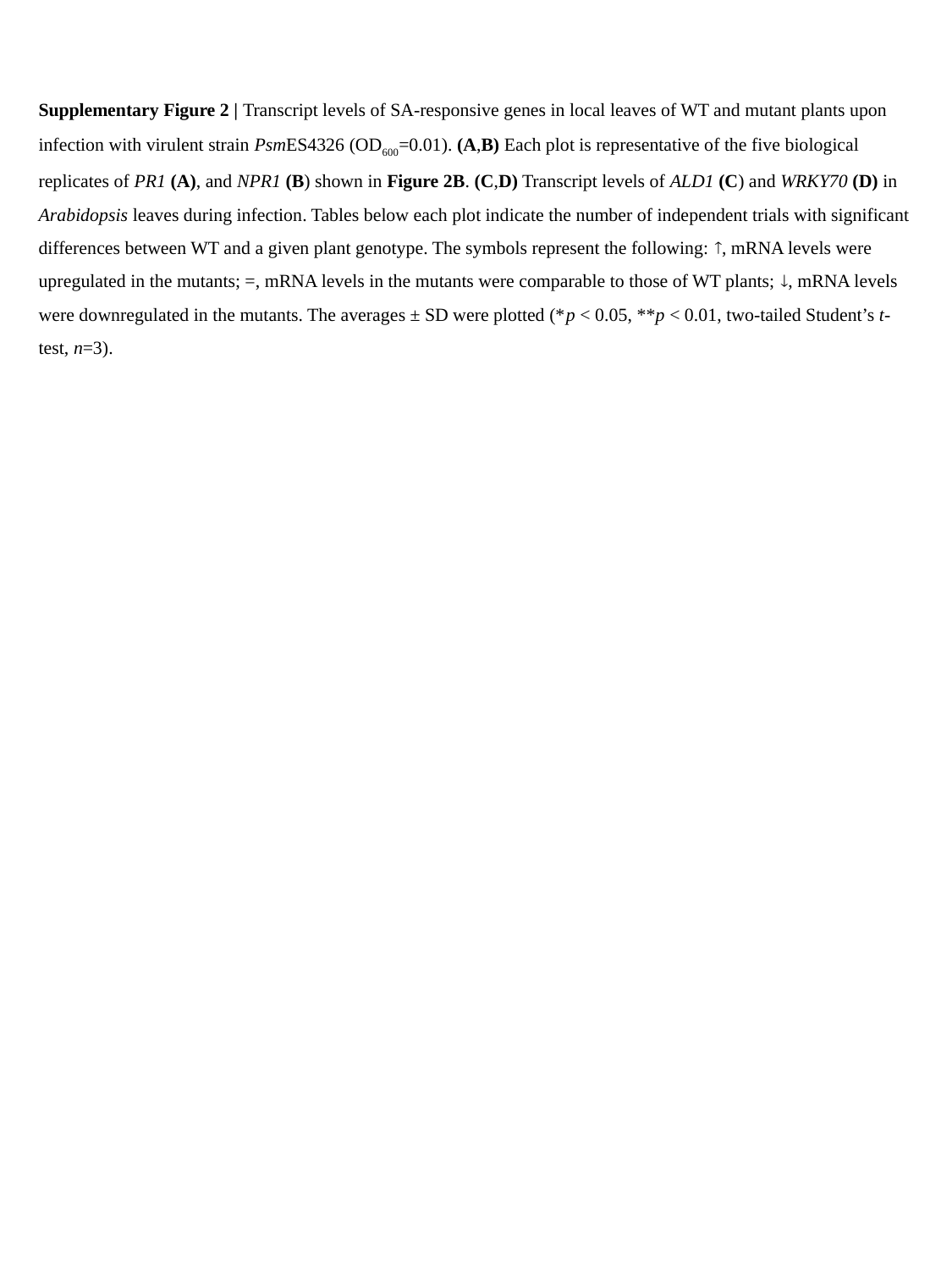

Supplementary Figure 2 | Transcript levels of SA-responsive genes in local leaves of WT and mutant plants upon infection with virulent strain PsmES4326 (OD600=0.01). (A,B) Each plot is representative of the five biological replicates of PR1 (A), and NPR1 (B) shown in Figure 2B. (C,D) Transcript levels of ALD1 (C) and WRKY70 (D) in Arabidopsis leaves during infection. Tables below each plot indicate the number of independent trials with significant differences between WT and a given plant genotype. The symbols represent the following: , mRNA levels were upregulated in the mutants; =, mRNA levels in the mutants were comparable to those of WT plants; , mRNA levels were downregulated in the mutants. The averages ± SD were plotted (*p < 0.05, **p < 0.01, two-tailed Student’s t-test, n=3).

## Slide 5
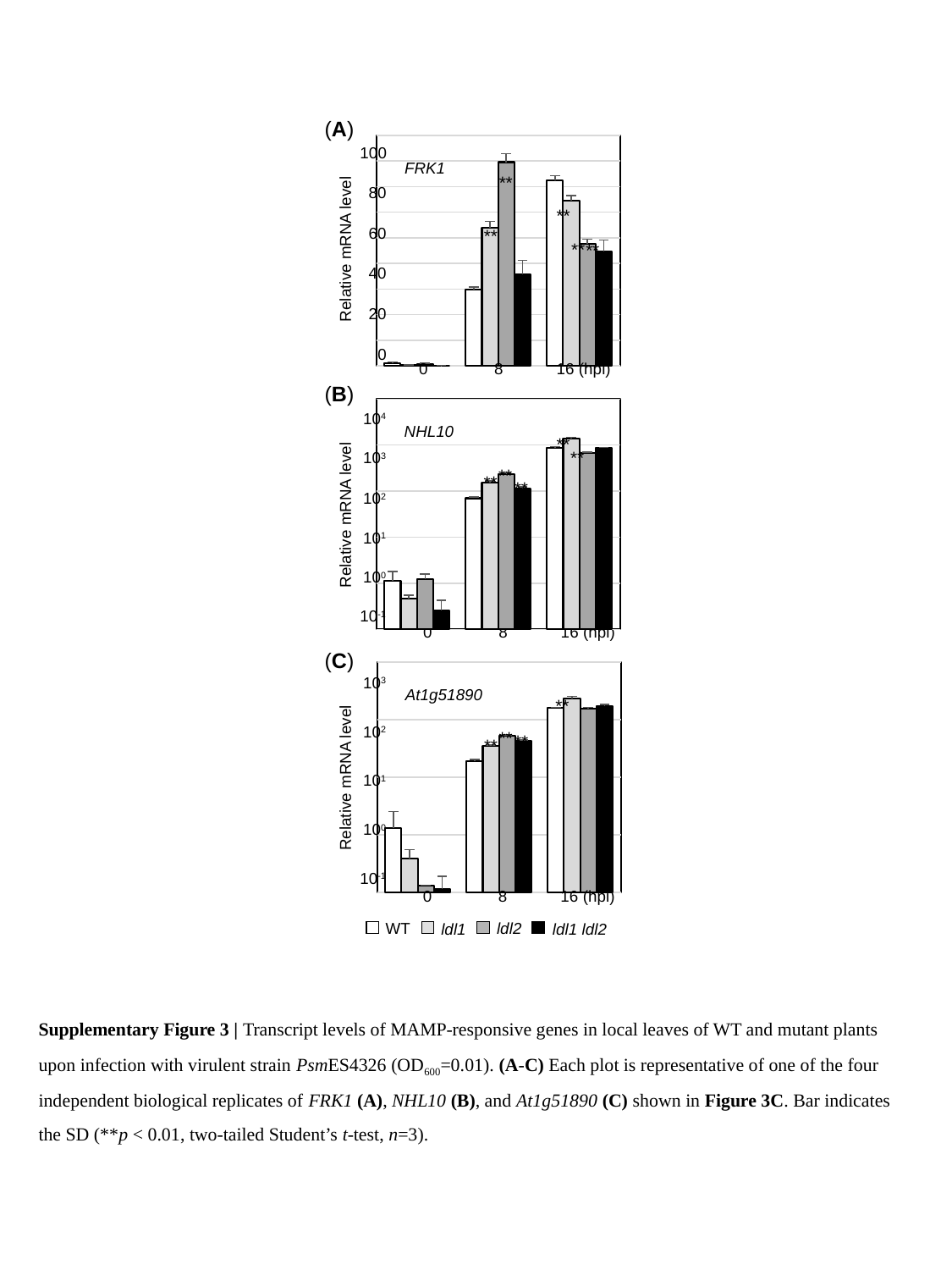

(A)
### Chart
| Category | | | | |
|---|---|---|---|---|100
FRK1
**
80
**
60
**
**
Relative mRNA level
**
40
20
0
 0 8 16 (hpi)
(B)
### Chart
| Category | | | | |
|---|---|---|---|---|104
NHL10
**
**
103
**
**
**
102
Relative mRNA level
101
100
10-1
 0 8 16 (hpi)
(C)
### Chart
| Category | | | | |
|---|---|---|---|---|103
At1g51890
**
102
**
**
**
Relative mRNA level
101
100
10-1
 0 8 16 (hpi)
WT
ldl2
ldl1
ldl1 ldl2
Supplementary Figure 3 | Transcript levels of MAMP-responsive genes in local leaves of WT and mutant plants upon infection with virulent strain PsmES4326 (OD600=0.01). (A-C) Each plot is representative of one of the four independent biological replicates of FRK1 (A), NHL10 (B), and At1g51890 (C) shown in Figure 3C. Bar indicates the SD (**p < 0.01, two-tailed Student’s t-test, n=3).

## Slide 6
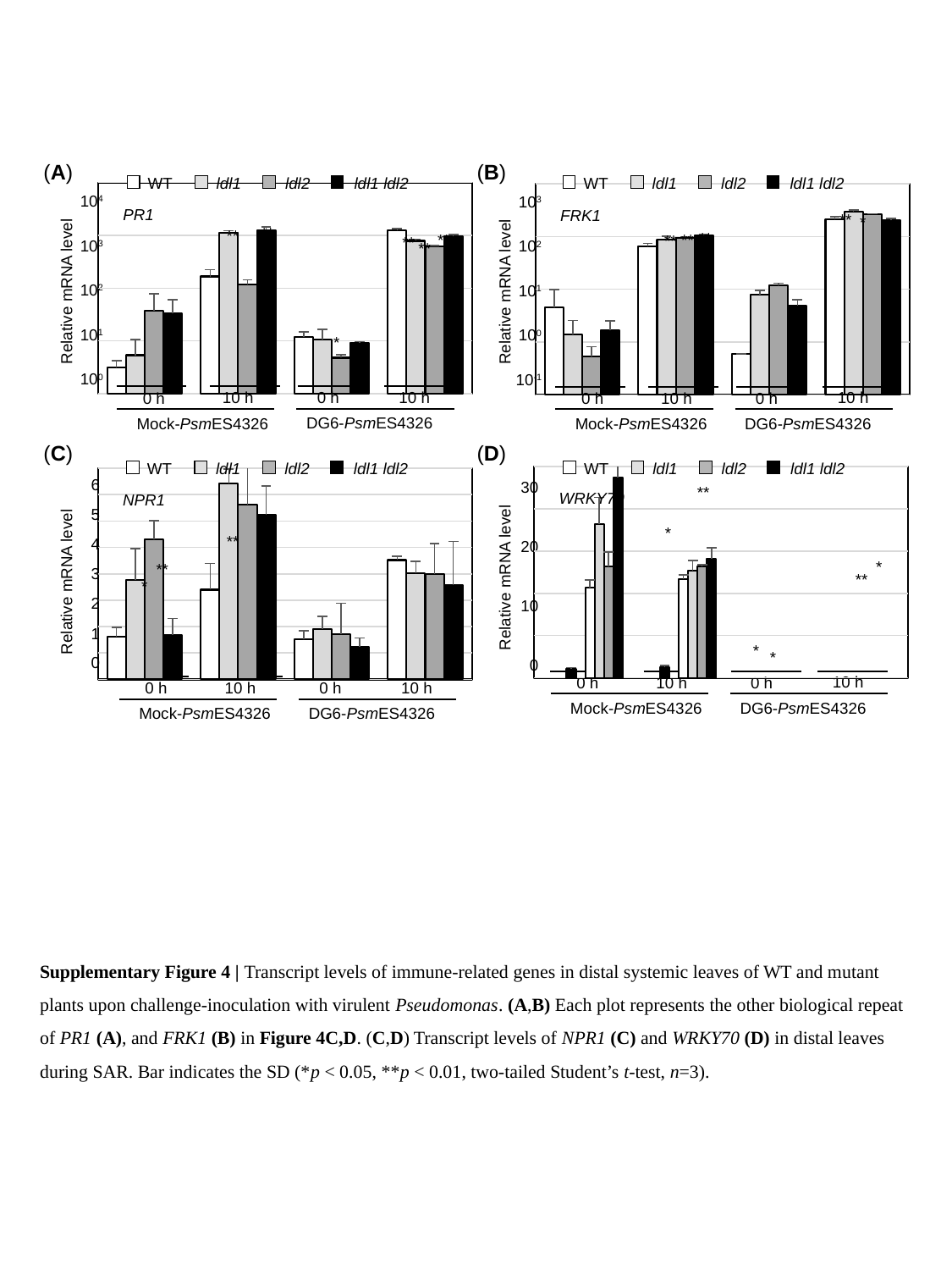

(A)
(B)
WT
ldl1
ldl2
ldl1 ldl2
WT
ldl1
ldl2
ldl1 ldl2
### Chart
| Category | 0 hours | 0 hours | 0 hours | 0 hours | 0 hours | 10 hours | 10 hours | 10 hours | 10 hours |
|---|---|---|---|---|---|---|---|---|---|
| MOCK | 3.113322687512498 | 5.360163578233745 | 37.87254997028862 | 32.898129021024616 | None | 166.66760161949222 | 1111.7838341106853 | 118.60461445419655 | 1259.3397285066924 |
| DG6 | 11.964458058291909 | 10.559449383247383 | 4.8136026552966555 | 9.240471385496214 | None | 1262.3383174086405 | 788.4315215245896 | 617.6714367218638 | 947.320377279798 |
### Chart
| Category | 0 hours | 0 hours | 0 hours | 0 hours | 0 hours | 10 hours | 10 hours | 10 hours | 10 hours |
|---|---|---|---|---|---|---|---|---|---|
| MOCK | 4.525883162223887 | 1.3982893852144862 | 0.5282671291763518 | 1.7002854278559572 | None | 64.94538813855232 | 86.51903456165577 | 94.24271055319616 | 103.16636051811015 |
| DG6 | 0.5973575675780094 | 7.97318876405826 | 11.71149770805017 | 4.827216305710457 | None | 211.25749681668216 | 298.47539659772946 | 261.49562923105054 | 202.6085047145441 |104
103
PR1
FRK1
**
*
**
**
**
**
**
**
**
103
102
**
102
Relative mRNA level
101
Relative mRNA level
101
100
*
100
10-1
10 h
0 h
10 h
10 h
0 h
0 h
10 h
0 h
DG6-PsmES4326
Mock-PsmES4326
DG6-PsmES4326
Mock-PsmES4326
(C)
(D)
WT
ldl1
ldl2
ldl1 ldl2
WT
ldl1
ldl2
ldl1 ldl2
### Chart
| Category | 0 hours | 0 hours | 0 hours | 0 hours | 0 hours | 10 hours | 10 hours | 10 hours | 10 hours |
|---|---|---|---|---|---|---|---|---|---|
| MOCK | 0.5051397360607572 | 1.4135218912529555 | 0.8167023050272993 | 1.0484424063325155 | None | 10.702272664480779 | 18.178698760509423 | 13.158381360661744 | 23.70629833340223 |
| DG6 | 0.9310677857514474 | 1.4641330156603363 | 0.6221265614404532 | 1.24662888727689 | None | 11.71852076974394 | 12.652505395820768 | 13.17857837037569 | 14.126505629809378 |
### Chart
| Category | 0 hours | 0 hours | 0 hours | 0 hours | 0 hours | 10 hours | 10 hours | 10 hours | 10 hours |
|---|---|---|---|---|---|---|---|---|---|
| MOCK | 0.8136484152055009 | 1.8802253325429163 | 2.655311101527842 | 0.8448354728628495 | None | 1.6982740838142123 | 3.7126241115151823 | 3.309971046892921 | 3.1112845858817995 |
| DG6 | 0.758258459835036 | 0.9553499510769612 | 0.8594278114335866 | 0.6163043031192083 | None | 2.2612965907442075 | 2.004873350265328 | 1.9882603889693964 | 1.785983105531382 |6
30
**
WRKY70
NPR1
5
*
**
4
20
*
*
**
3
Relative mRNA level
**
Relative mRNA level
*
2
10
1
*
*
0
0
10 h
0 h
10 h
0 h
10 h
0 h
10 h
0 h
DG6-PsmES4326
Mock-PsmES4326
DG6-PsmES4326
Mock-PsmES4326
Supplementary Figure 4 | Transcript levels of immune-related genes in distal systemic leaves of WT and mutant plants upon challenge-inoculation with virulent Pseudomonas. (A,B) Each plot represents the other biological repeat of PR1 (A), and FRK1 (B) in Figure 4C,D. (C,D) Transcript levels of NPR1 (C) and WRKY70 (D) in distal leaves during SAR. Bar indicates the SD (*p < 0.05, **p < 0.01, two-tailed Student’s t-test, n=3).

## Slide 7
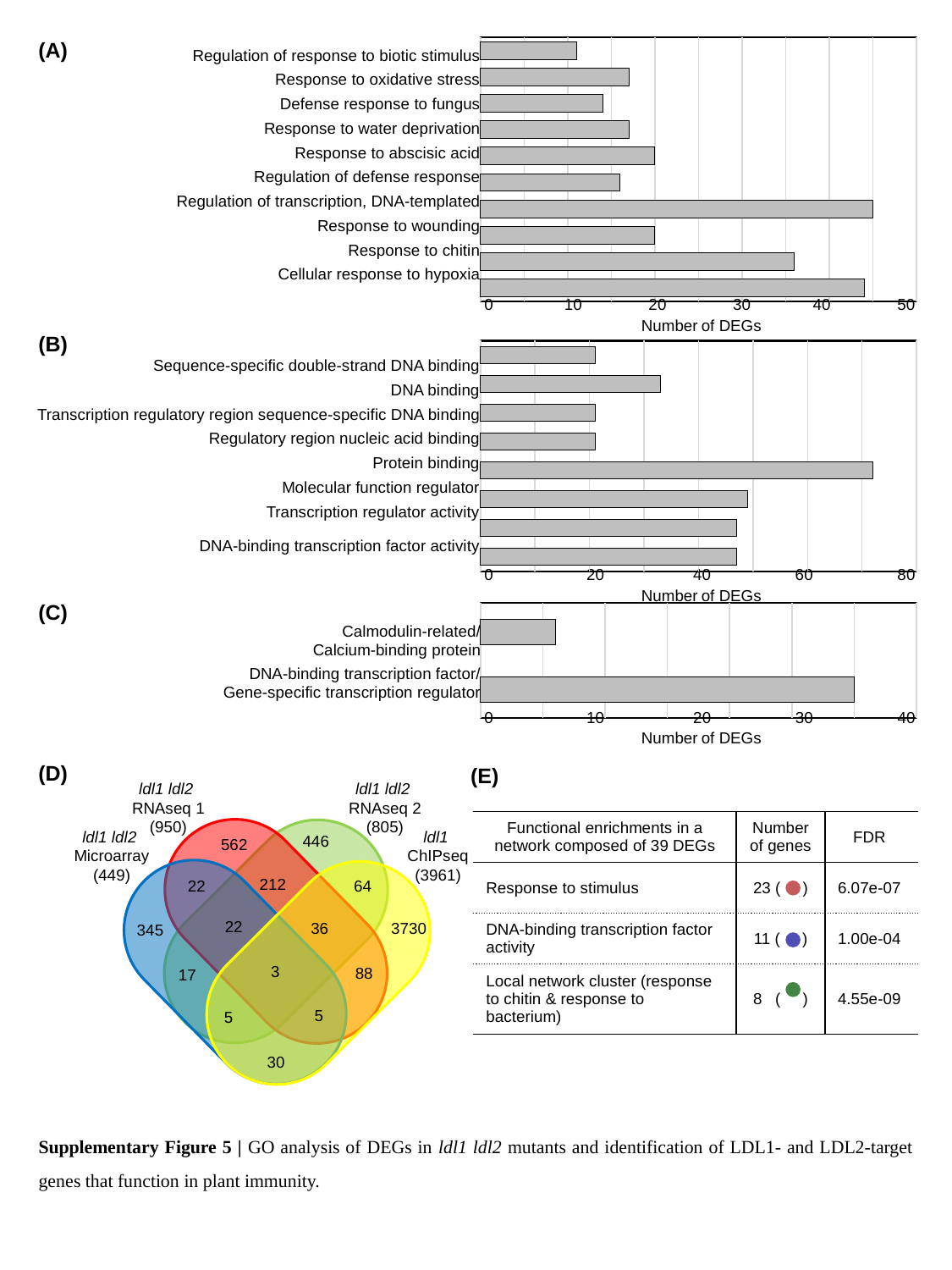

(A)
### Chart
| Category | |
|---|---|
| cellular response to hypoxia | 44.0 |
| response to chitin | 36.0 |
| response to wounding | 20.0 |
| regulation of transcription, DNA-templated | 45.0 |
| regulation of defense response | 16.0 |
| response to abscisic acid | 20.0 |
| response to water deprivation | 17.0 |
| defense response to fungus | 14.0 |
| response to oxidative stress | 17.0 |
| regulation of response to biotic stimulus | 11.0 |Regulation of response to biotic stimulus
Response to oxidative stress
Defense response to fungus
Response to water deprivation
Response to abscisic acid
Regulation of defense response
Regulation of transcription, DNA-templated
Response to wounding
Response to chitin
Cellular response to hypoxia
0 10 20 30 40 50
Number of DEGs
(B)
### Chart
| Category | |
|---|---|
| DNA-binding transcription factor activity | 47.0 |
| transcription regulator activity | 47.0 |
| molecular function regulator | 49.0 |
| protein binding | 72.0 |
| regulatory region nucleic acid binding | 21.0 |
| transcription regulatory region sequence-specific DNA binding | 21.0 |
| DNA binding | 33.0 |
| sequence-specific double-stranded DNA binding | 21.0 |Sequence-specific double-strand DNA binding
DNA binding
Transcription regulatory region sequence-specific DNA binding
Regulatory region nucleic acid binding
Protein binding
Molecular function regulator
Transcription regulator activity
DNA-binding transcription factor activity
0 20 40 60 80
Number of DEGs
(C)
### Chart
| Category | |
|---|---|
| gene-specific transcriptional regulator | 30.0 |
| calmodulin-related | 6.0 |Calmodulin-related/
Calcium-binding protein
DNA-binding transcription factor/
Gene-specific transcription regulator
0 10 20 30 40
Number of DEGs
(D)
(E)
ldl1 ldl2
RNAseq 1
(950)
ldl1 ldl2
RNAseq 2
(805)
ldl1 ldl2
Microarray
(449)
ldl1
ChIPseq
(3961)
446
562
212
22
64
22
3730
36
345
3
88
17
5
5
30
| Functional enrichments in a network composed of 39 DEGs | Number of genes | FDR |
| --- | --- | --- |
| Response to stimulus | 23 ( ) | 6.07e-07 |
| DNA-binding transcription factor activity | 11 ( ) | 1.00e-04 |
| Local network cluster (response to chitin & response to bacterium) | 8 ( ) | 4.55e-09 |
Supplementary Figure 5 | GO analysis of DEGs in ldl1 ldl2 mutants and identification of LDL1- and LDL2-target genes that function in plant immunity.

## Slide 8
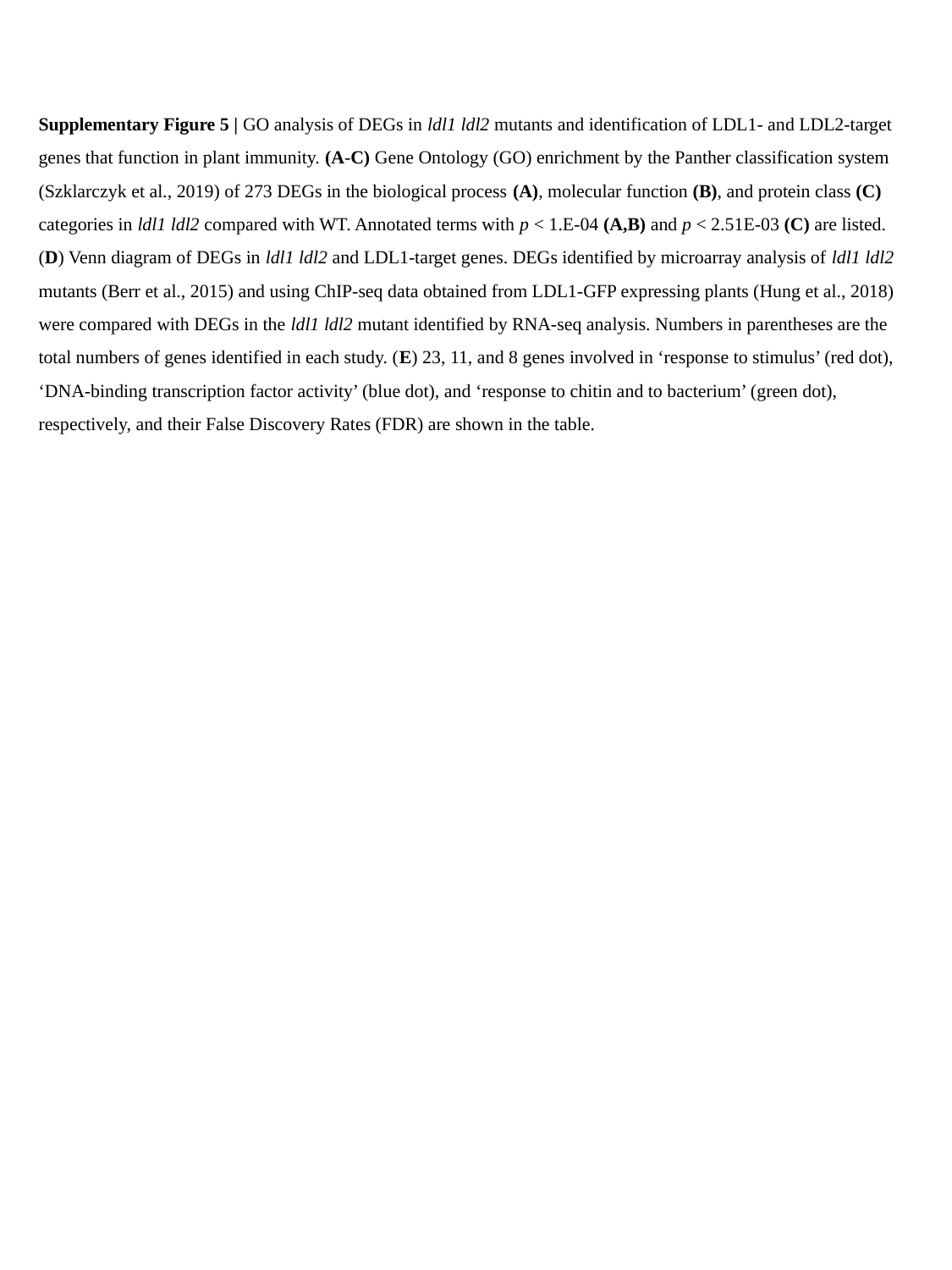

Supplementary Figure 5 | GO analysis of DEGs in ldl1 ldl2 mutants and identification of LDL1- and LDL2-target genes that function in plant immunity. (A-C) Gene Ontology (GO) enrichment by the Panther classification system (Szklarczyk et al., 2019) of 273 DEGs in the biological process (A), molecular function (B), and protein class (C) categories in ldl1 ldl2 compared with WT. Annotated terms with p < 1.E-04 (A,B) and p < 2.51E-03 (C) are listed. (D) Venn diagram of DEGs in ldl1 ldl2 and LDL1-target genes. DEGs identified by microarray analysis of ldl1 ldl2 mutants (Berr et al., 2015) and using ChIP-seq data obtained from LDL1-GFP expressing plants (Hung et al., 2018) were compared with DEGs in the ldl1 ldl2 mutant identified by RNA-seq analysis. Numbers in parentheses are the total numbers of genes identified in each study. (E) 23, 11, and 8 genes involved in ‘response to stimulus’ (red dot), ‘DNA-binding transcription factor activity’ (blue dot), and ‘response to chitin and to bacterium’ (green dot), respectively, and their False Discovery Rates (FDR) are shown in the table.

## Slide 9
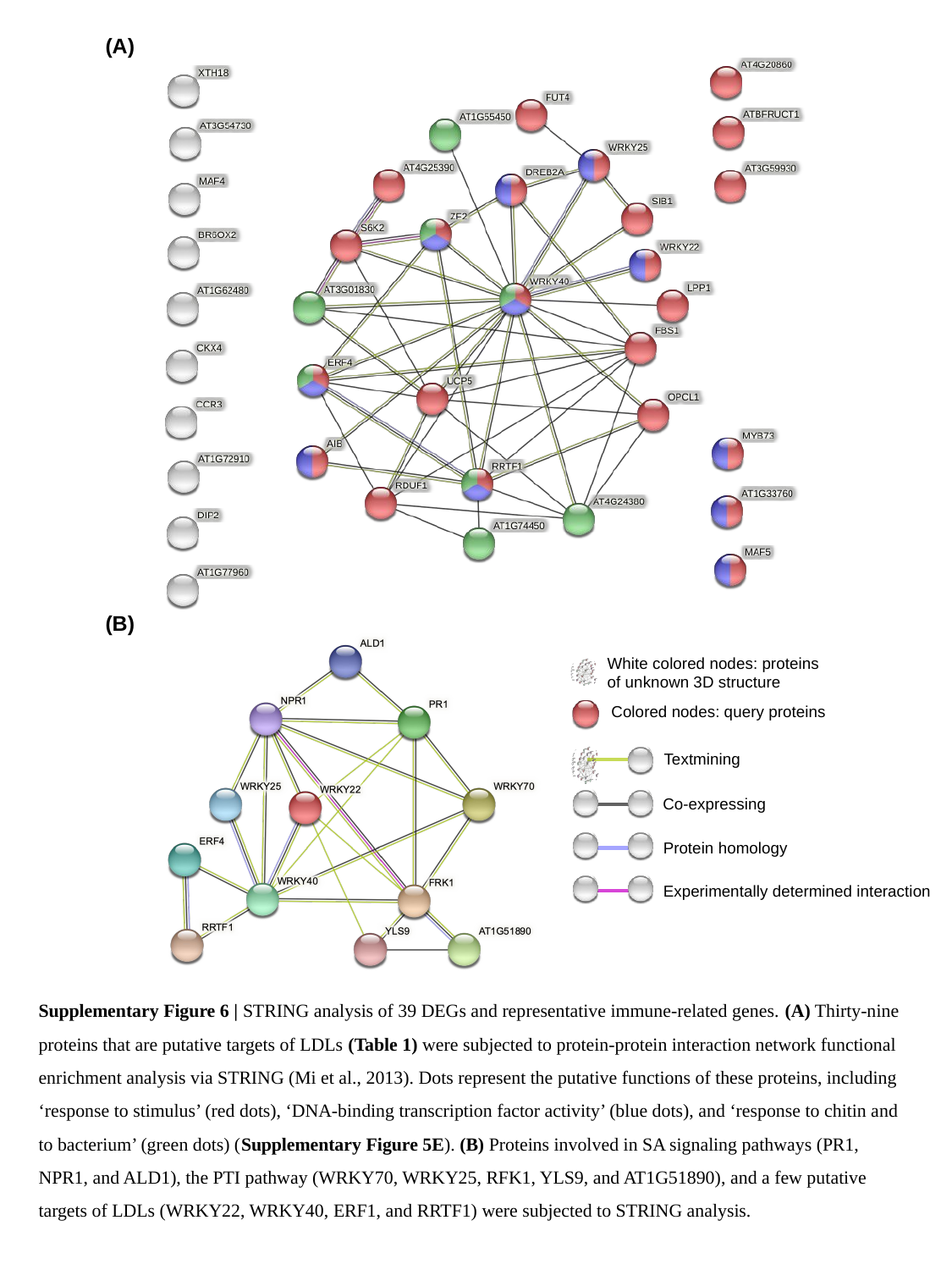

(A)
(B)
White colored nodes: proteins of unknown 3D structure
Colored nodes: query proteins
Textmining
Co-expressing
Protein homology
Experimentally determined interaction
Supplementary Figure 6 | STRING analysis of 39 DEGs and representative immune-related genes. (A) Thirty-nine proteins that are putative targets of LDLs (Table 1) were subjected to protein-protein interaction network functional enrichment analysis via STRING (Mi et al., 2013). Dots represent the putative functions of these proteins, including ‘response to stimulus’ (red dots), ‘DNA-binding transcription factor activity’ (blue dots), and ‘response to chitin and to bacterium’ (green dots) (Supplementary Figure 5E). (B) Proteins involved in SA signaling pathways (PR1, NPR1, and ALD1), the PTI pathway (WRKY70, WRKY25, RFK1, YLS9, and AT1G51890), and a few putative targets of LDLs (WRKY22, WRKY40, ERF1, and RRTF1) were subjected to STRING analysis.

## Slide 10
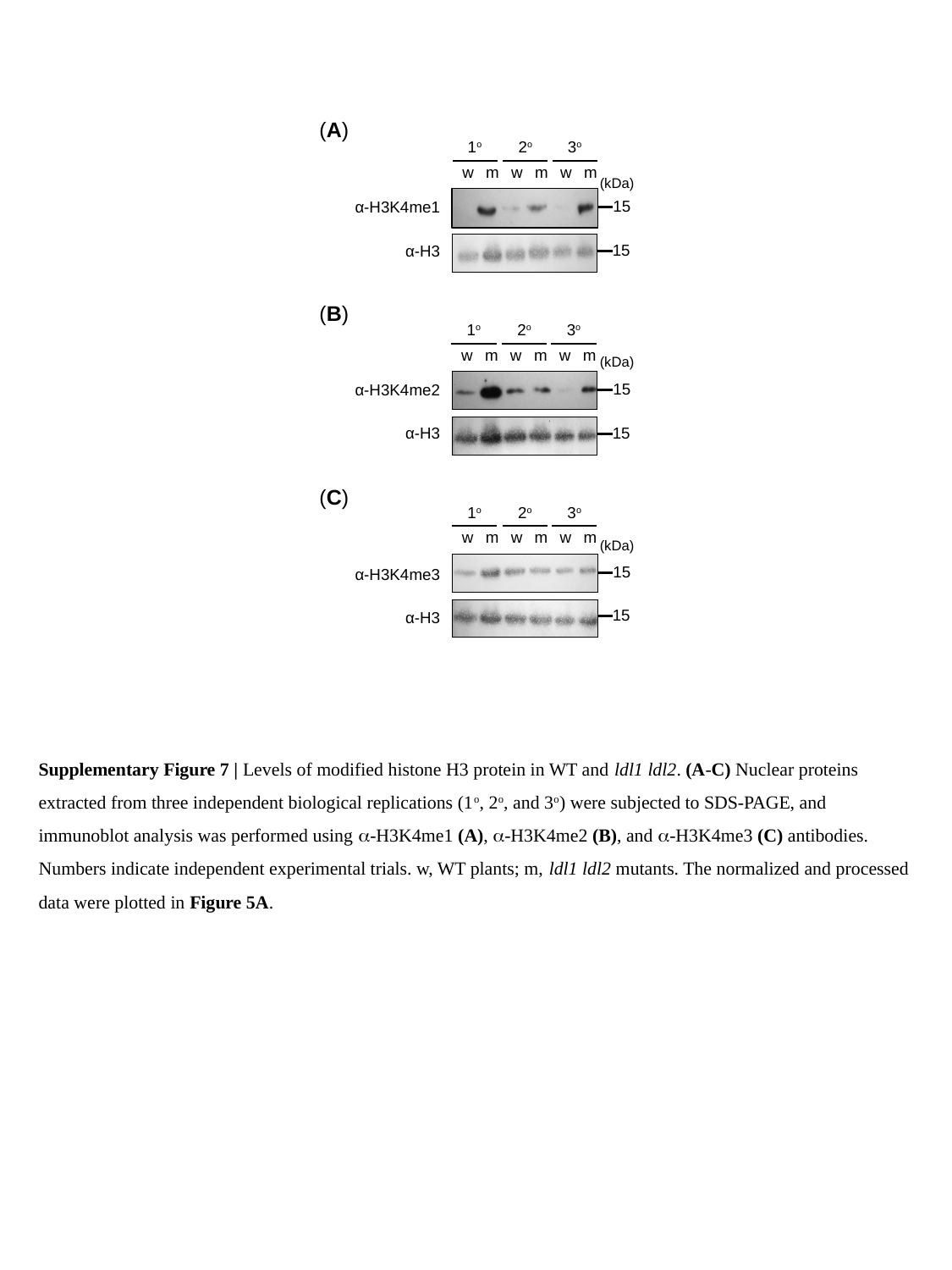

(A)
1o
2o
3o
w
m
w
m
w
m
15
15
α-H3K4me1
α-H3
(B)
1o
2o
3o
w
m
w
m
w
m
15
15
α-H3K4me2
α-H3
(C)
1o
2o
3o
w
m
w
m
w
m
15
15
α-H3K4me3
α-H3
(kDa)
(kDa)
(kDa)
Supplementary Figure 7 | Levels of modified histone H3 protein in WT and ldl1 ldl2. (A-C) Nuclear proteins extracted from three independent biological replications (1o, 2o, and 3o) were subjected to SDS-PAGE, and immunoblot analysis was performed using α-H3K4me1 (A), α-H3K4me2 (B), and α-H3K4me3 (C) antibodies. Numbers indicate independent experimental trials. w, WT plants; m, ldl1 ldl2 mutants. The normalized and processed data were plotted in Figure 5A.

## Slide 11
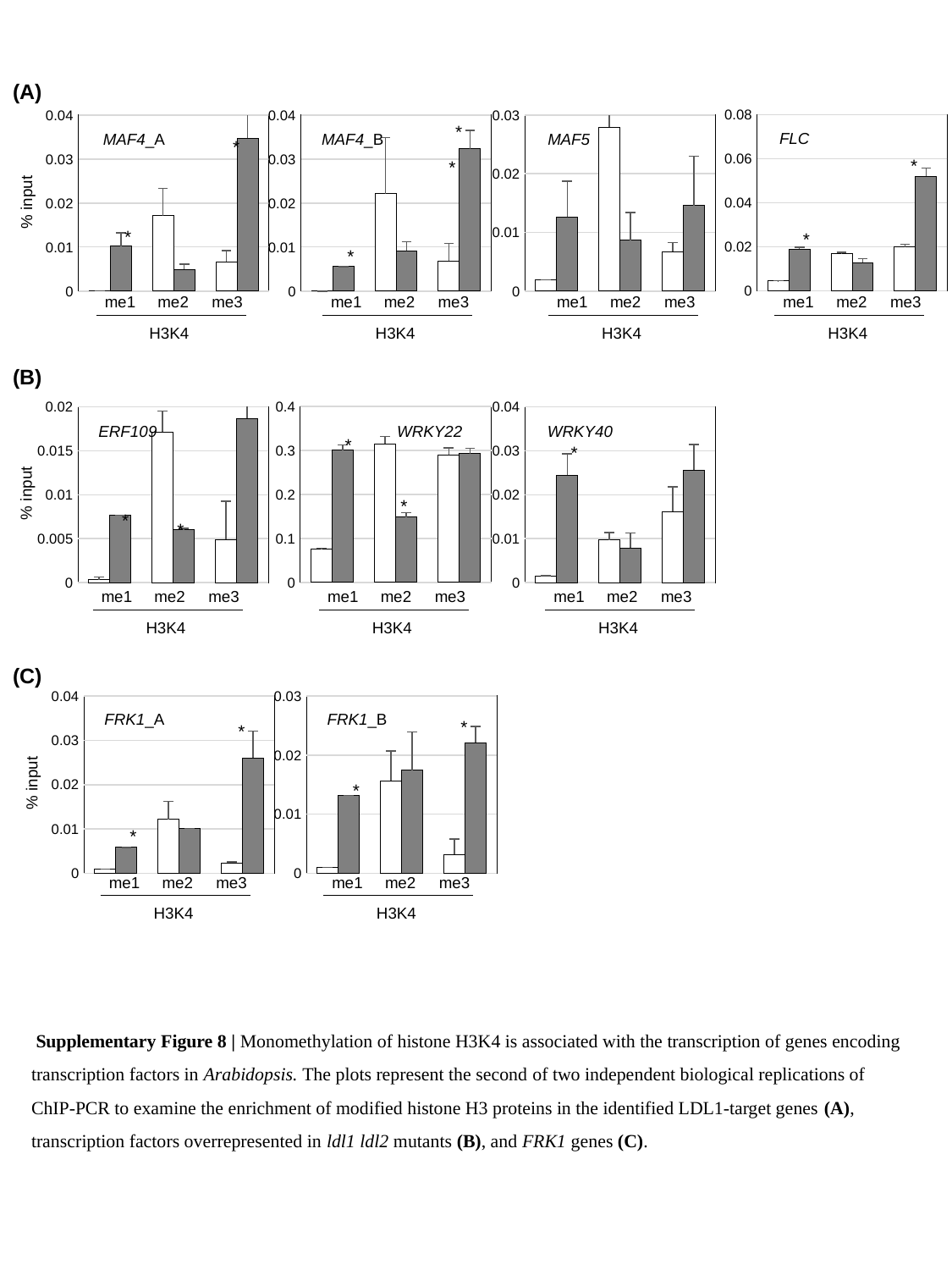

(A)
### Chart
| Category | WT | ldl1 ldl2 |
|---|---|---|
| me1 | 0.004483588115501505 | 0.01882786897875124 |
| me2 | 0.016812846864086975 | 0.012679726669282161 |
| me3 | 0.019831400555819723 | 0.05196651059589546 |
### Chart
| Category | WT | ldl1 ldl2 |
|---|---|---|
| me1 | 1.2933999603565486e-06 | 0.01022633101576696 |
| me2 | 0.017165440092549424 | 0.004945014923341235 |
| me3 | 0.006662434094849225 | 0.03471256913954799 |
### Chart
| Category | WT | ldl1 ldl2 |
|---|---|---|
| me1 | 0.0 | 0.005554739906706618 |
| me2 | 0.022141416528692767 | 0.0090908455176041 |
| me3 | 0.006794664640056279 | 0.03235578179998371 |
### Chart
| Category | WT | ldl1 ldl2 |
|---|---|---|
| me1 | 0.0019304978234885339 | 0.012606694783667442 |
| me2 | 0.02793906893968585 | 0.008687363711981592 |
| me3 | 0.006729738544777361 | 0.014540509529171469 |*
FLC
MAF4_A
MAF4_B
MAF5
*
*
*
% input
*
*
*
me1
me2
me3
H3K4
me1
me2
me3
H3K4
me1
me2
me3
H3K4
me1
me2
me3
H3K4
(B)
### Chart
| Category | WT | ldl1 ldl2 |
|---|---|---|
| me1 | 0.075104474702009 | 0.29999344381334686 |
| me2 | 0.3149055759004052 | 0.1479322065362135 |
| me3 | 0.28976960596620555 | 0.2924935177123896 |
### Chart
| Category | WT | ldl1 ldl2 |
|---|---|---|
| me1 | 0.00031726499149763187 | 0.007688256075301207 |
| me2 | 0.017133533137398852 | 0.005986452473980285 |
| me3 | 0.004875264983702882 | 0.018671068176313258 |
### Chart
| Category | WT | ldl1 ldl2 |
|---|---|---|
| me1 | 0.001395059257117055 | 0.02440046162010001 |
| me2 | 0.009661936065659084 | 0.007902955140199041 |
| me3 | 0.016182383333000868 | 0.02562793508097838 |ERF109
WRKY22
WRKY40
*
*
% input
*
*
*
me1
me2
me3
H3K4
me1
me2
me3
H3K4
me1
me2
me3
H3K4
(C)
### Chart
| Category | WT | ldl1 ldl2 |
|---|---|---|
| me1 | 0.001 | 0.005878850121053344 |
| me2 | 0.012195474998426298 | 0.010071562003225298 |
| me3 | 0.0023065679818394208 | 0.026044210822807178 |
### Chart
| Category | WT | ldl1 ldl2 |
|---|---|---|
| me1 | 0.001 | 0.013176843132870798 |
| me2 | 0.015630304412114063 | 0.017407219570542354 |
| me3 | 0.003213946816493033 | 0.022053561478036696 |FRK1_A
FRK1_B
*
*
% input
*
*
me1
me2
me3
H3K4
me1
me2
me3
H3K4
 Supplementary Figure 8 | Monomethylation of histone H3K4 is associated with the transcription of genes encoding transcription factors in Arabidopsis. The plots represent the second of two independent biological replications of ChIP-PCR to examine the enrichment of modified histone H3 proteins in the identified LDL1-target genes (A), transcription factors overrepresented in ldl1 ldl2 mutants (B), and FRK1 genes (C).

## Slide 12
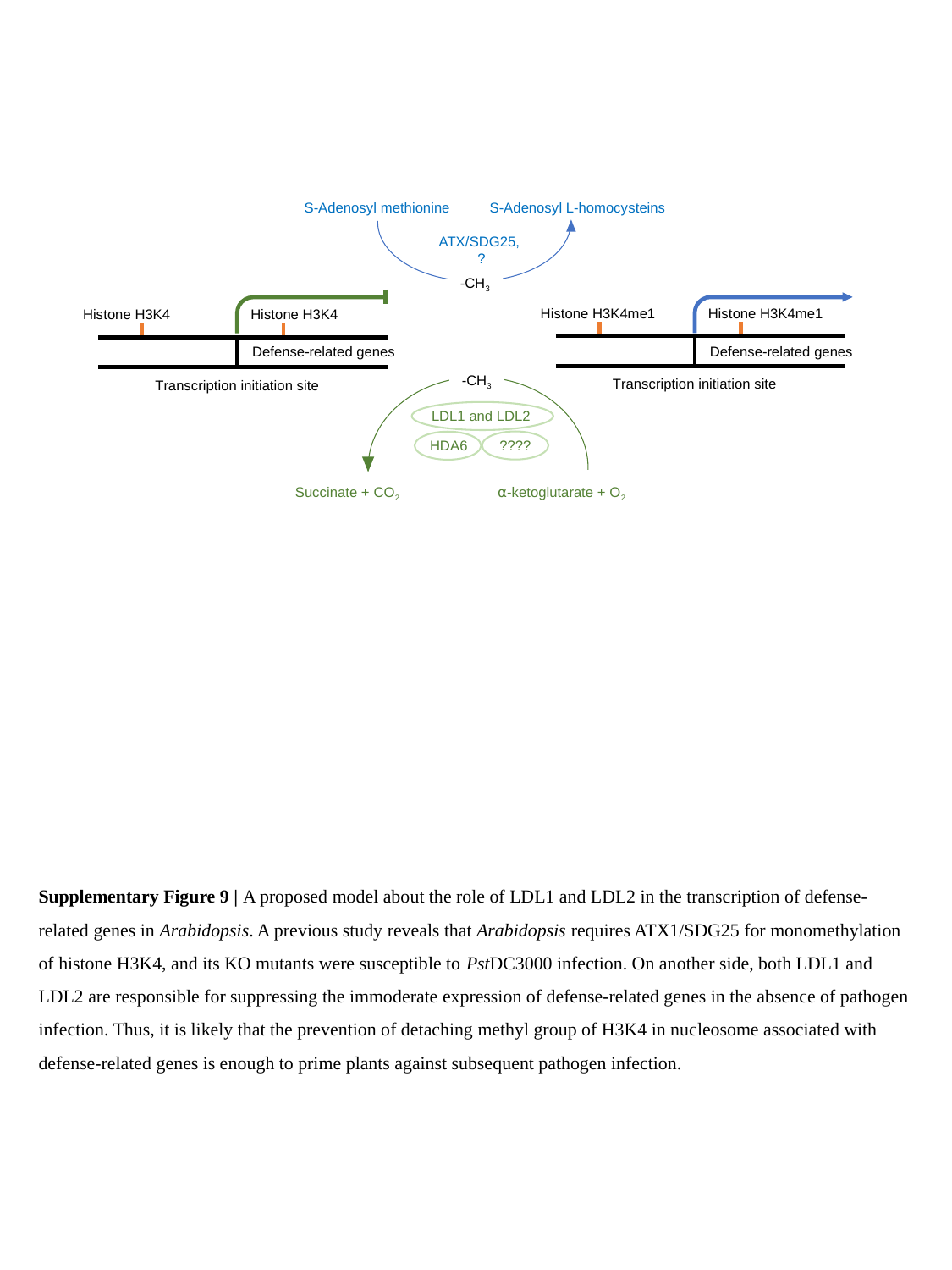

S-Adenosyl methionine
S-Adenosyl L-homocysteins
ATX/SDG25,
?
-CH3
Histone H3K4me1
Histone H3K4me1
Histone H3K4
Histone H3K4
Defense-related genes
Defense-related genes
-CH3
Transcription initiation site
Transcription initiation site
LDL1 and LDL2
HDA6
????
Succinate + CO2
⍺-ketoglutarate + O2
Supplementary Figure 9 | A proposed model about the role of LDL1 and LDL2 in the transcription of defense-related genes in Arabidopsis. A previous study reveals that Arabidopsis requires ATX1/SDG25 for monomethylation of histone H3K4, and its KO mutants were susceptible to PstDC3000 infection. On another side, both LDL1 and LDL2 are responsible for suppressing the immoderate expression of defense-related genes in the absence of pathogen infection. Thus, it is likely that the prevention of detaching methyl group of H3K4 in nucleosome associated with defense-related genes is enough to prime plants against subsequent pathogen infection.
